# Decoding early lung adenocarcinoma progression by single-cell and spatial transcriptomics reveals a CMA-related prognostic signature

**DOI:** 10.3389/fimmu.2026.1875096

**Published:** 2026-07-09

**Authors:** Junkang Wang, Wenxuan Wang, Shengnan Li, Xinyu Ji

**Affiliations:** 1 Department of Neurosurgery, The First Affiliated Hospital of China Medical University, Shenyang, China; 2 Department of Pain and Rehabilitation, Liaoning Cancer Hospital & Institute, Shenyang, China; 3 Department of Thoracic Surgery, The First Affiliated Hospital of China Medical University, Shenyang, China

**Keywords:** chaperone-mediated autophagy, lung adenocarcinoma, machine learning, prognostic signature, single-cell RNA sequencing, spatial transcriptomics, tumor microenvironment

## Abstract

**Background:**

Lung adenocarcinoma (LUAD) progression from adenocarcinoma *in situ* (AIS) to minimally invasive adenocarcinoma (MIA) and invasive adenocarcinoma (IAC) is accompanied by molecular heterogeneity and tumor microenvironment remodeling. Chaperone-mediated autophagy (CMA) regulates tumor cell homeostasis, metabolic adaptation, and stress responses, but its dynamic alterations and prognostic significance during the AIS/MIA-to-IAC progression of LUAD remain unclear.

**Methods:**

We integrated the single-cell transcriptomic dataset GSE189357 and the spatial transcriptomic dataset GSE189487 with bulk transcriptomic data from TCGA-LUAD, GTEx, and the GEO validation cohorts GSE31210 and GSE50081 to characterize CMA-related features during the AIS/MIA-to-IAC progression of LUAD. CMA activity and myeloid remodeling were analyzed at the single-cell and spatial levels. Candidate genes were identified by combining tumor-normal differential expression analysis in TCGA-LUAD with weighted gene co-expression network analysis. Multiple machine learning algorithms were compared to construct and externally validate a prognostic model. Biological and clinical relevance was further assessed through clinicopathological, pathway, immune, cell-cell communication, drug sensitivity, and *in vitro* analyses.

**Results:**

CMA-related activity showed marked cell-type specificity and spatial heterogeneity during the AIS/MIA-to-IAC progression of LUAD, with the most prominent changes in the myeloid compartment. Myeloid re-clustering revealed enrichment of cDC2 and APOE+ lipid-associated TAMs in IAC, whereas FABP4+ metabolic TAMs and immature neutrophils decreased. By integrating tumor-normal differential expression analysis with weighted gene co-expression network analysis, 122 candidate genes were identified, and a 15-gene CMA-related prognostic signature was established using a random survival forest model. This signature showed robust prognostic stratification in TCGA-LUAD, GSE31210, and GSE50081. The high-risk group had poorer survival, more advanced stage, and enrichment of malignant pathways including GLYCOLYSIS, G2M CHECKPOINT, MTORC1 SIGNALING, E2F TARGETS, and MYC TARGETS. The low-risk group showed higher stromal and immune scores and stronger immune activity. THBS1 signaling was restricted to high-risk epithelial communication, with fibroblasts as the major signal senders. *In vitro* experiments showed that MGP overexpression inhibited lung cancer cell proliferation, colony formation, migration, and invasion.

**Conclusions:**

This study characterized CMA-related heterogeneity during LUAD progression from AIS to IAC and established a robust 15-gene prognostic signature. Fibroblast-derived THBS1 signaling and MGP may contribute to the high-risk phenotype and provide insight into early LUAD evolution and risk stratification.

## 1 Introduction

Lung cancer remains one of the leading causes of cancer incidence and mortality worldwide, and non-small cell lung cancer (NSCLC) accounts for approximately 85% of all cases (1). Among these, lung adenocarcinoma (LUAD) is the most common histological subtype (2, 3). Despite continuous advances in screening, surgery, targeted therapy, and immunotherapy, the clinical outcomes of patients with LUAD remain markedly heterogeneous. In particular, during early pathological progression, the transition from adenocarcinoma *in situ* (AIS) to minimally invasive adenocarcinoma (MIA) and then to invasive adenocarcinoma (IAC) is not simply driven by an increase in tumor cell number but is accompanied by continuous remodeling of tumor cell intrinsic states, the immune microenvironment, and intercellular interaction networks (4–6). Therefore, identifying key molecular programs associated with the AIS/MIA-to-IAC progression of LUAD and establishing robust prognostic models that reflect its biological heterogeneity are of substantial importance for improving risk stratification and guiding individualized treatment.

Chaperone-mediated autophagy (CMA) is a selective lysosomal degradation pathway that plays a crucial role in maintaining protein homeostasis, metabolic adaptation, and stress responses (7, 8). Increasing evidence has shown that CMA exerts context-dependent effects in cancer (9). In some tumor types, CMA activation facilitates tumor cell adaptation to oxidative stress, metabolic pressure, and therapeutic stimuli, thereby promoting tumor progression (10). In other settings, CMA-related molecules may be associated with cellular homeostasis and suppression of malignant phenotypes (11). Studies focusing on the core CMA receptor LAMP2A have further suggested that its aberrant expression is linked to poor prognosis in multiple cancers and has also been associated with clinical outcome and treatment response in lung cancer (12, 13). These observations indicate that CMA is not merely a metabolism-related process, but may be deeply involved in the dynamic balance between tumor cells and the microenvironment (14).

Meanwhile, advances in single-cell RNA sequencing and spatial transcriptomics have provided new opportunities to dissect cellular heterogeneity and microenvironmental remodeling during the AIS/MIA-to-IAC progression of LUAD (15, 16). Previous studies integrating single-cell and spatial transcriptomic data have shown that AIS, MIA, and IAC are associated with substantial multicellular compositional changes, spatial reprogramming, and altered intercellular communication, suggesting that the AIS/MIA-to-IAC progression of LUAD is fundamentally a process of continuous remodeling of the multicellular ecosystem (17, 18). In particular, the early LUAD single-cell and spatial atlas constructed by Zhu et al. indicated that epithelial tumor evolution is tightly coupled with changes in immune and stromal cell states (19). However, current studies on CMA-related features in LUAD have largely remained at the bulk level or focused on individual genes, and there is still a lack of systematic integration of single-cell, spatial, and bulk transcriptomic data combined with prognostic modeling and functional validation. In other words, although existing work has shown that early LUAD undergoes complex remodeling, it remains unclear how CMA-related programs are distributed during this process, which cellular compartments drive these changes, and whether such alterations can be translated into a stable and biologically interpretable prognostic stratification tool.

Based on this background, we integrated LUAD single-cell transcriptomic, spatial transcriptomic, and large-scale bulk transcriptomic datasets to systematically characterize the heterogeneous distribution of CMA-related features during the AIS/MIA-to-IAC progression of LUAD, with a particular focus on dynamic remodeling of the myeloid compartment. On this basis, we combined differential expression analysis, weighted gene co-expression network analysis, and multiple machine learning algorithms to identify key genes and construct a robust CMA-related prognostic signature. Furthermore, we evaluated the biological and clinical significance of this signature from multiple perspectives, including clinicopathological features, pathway activity, immune microenvironment, single-cell communication, and drug sensitivity, and further validated the tumor-suppressive role of the core gene MGP through *in vitro* experiments. This study may provide a new perspective for understanding the molecular evolution of LUAD from AIS to IAC and offer a reference for risk-stratified prognostic assessment and therapeutic decision-making.

## 2 Materials and methods

### 2.1 Data collection

This study integrated single-cell transcriptomic, spatial transcriptomic, bulk transcriptomic, and *in vitro* experimental data to systematically evaluate the heterogeneity of chaperone-mediated autophagy (CMA)-related features during early pathological progression of lung adenocarcinoma (LUAD) and to construct a CMA-related prognostic model. Single-cell RNA sequencing data were obtained from the GEO dataset GSE189357, which included 9 LUAD samples, comprising 3 AIS samples (TD5, TD7, TD8), 3 MIA samples (TD3, TD4, TD6), and 3 IAC samples (TD1, TD2, TD9). Spatial transcriptomic data were obtained from GSE189487, which included 6 samples, comprising 2 AIS samples (TD5, TD8), 2 MIA samples (TD3, TD6), and 2 IAC samples (TD1, TD2). Bulk transcriptomic datasets included TCGA-LUAD, GTEx normal lung tissues, and the GEO validation cohorts GSE31210 and GSE50081. TCGA-LUAD contained 600 tumor samples and 59 adjacent normal samples, while GTEx included 578 normal lung tissue samples. GSE31210 and GSE50081 contained 226 and 127 LUAD samples with survival information, respectively.

### 2.2 Single-cell and spatial transcriptomic analyses

Single-cell RNA sequencing data were processed and analyzed using Seurat (20). Raw expression matrices were first quality controlled to remove low-quality cells and lowly expressed genes. The retained cells were then normalized, followed by identification of highly variable genes, data scaling, and principal component analysis. Clustering was performed based on the principal components, and Uniform Manifold Approximation and Projection (UMAP) was used for dimensionality reduction and visualization. Major cell populations were annotated according to canonical marker genes, resulting in the identification of Epithelial, T/NK cells, Myeloid, B cells, Plasma cells, Mast cells, Endothelial, Fibroblasts, and Proliferating cells. Cells were further grouped into AIS, MIA, and IAC according to pathological stage for downstream comparisons.

For spatial transcriptomic analysis, single-cell data were used as a reference for cell type deconvolution of spatial spots using RCTD (Robust Cell Type Decomposition) implemented in the spacexr package. The analysis was performed with max_cores = 8 and doublet_mode = “full”. Cell type names were first unified, after which a reference object was constructed and integrated with spot-level expression matrices, coordinate information, and UMI counts to infer spot-level cell-type composition. Importantly, both the single-cell and spatial transcriptomic datasets were derived from human LUAD samples from the same institution and the same published study (4), which improved the biological comparability between the reference and query datasets. In addition, the inferred spatial distributions of major cell populations were broadly consistent with the spatial expression patterns of representative marker genes in the corresponding sections. The spatial enrichment of major cell types and the spatial distribution of CMA scores were then visualized to assess stage-specific heterogeneity in cellular composition and CMA activity.

### 2.3 Data preprocessing

Bulk transcriptomic data were uniformly preprocessed before analysis. Common genes across the TCGA-LUAD, GSE31210, and GSE50081 cohorts were first extracted, and corresponding clinical follow-up information was integrated. For raw count or TPM-format expression data, log2(x + 1) transformation was applied to reduce the influence of extreme values. Expression matrices from the three cohorts were then merged, and batch effects were corrected simultaneously across all cohorts using the ComBat method, with cohort identity specified as the batch variable, to minimize systematic bias introduced by different platforms and data sources (21). All gene expression values were further standardized before model construction to improve comparability and stability across cohorts.

Samples with missing key follow-up information, including overall survival time or survival status, were excluded before analysis. Overall survival was defined as the time from initial diagnosis to death or last follow-up, and survival status was coded as dead or censored.

### 2.4 CMA score calculation and myeloid cell re-clustering

To assess CMA activity across different cell populations and spatial regions, a predefined CMA-related gene set was curated and classified into three categories: effector genes (n = 26), positive regulators (n = 43), and negative regulators (n = 15). The full gene list is provided in **Supplementary Table 1**. For both single-cell and spatial transcriptomic analyses, the CMA score was calculated as: CMA score = (2 × sum of effector gene expression + sum of positive regulator gene expression − sum of negative regulator gene expression)/84. A higher weight was assigned to effector genes because they represent the core execution machinery directly involved in the autophagy process, whereas positive and negative regulators mainly reflect upstream regulatory influences on pathway activity.

Given that myeloid cells exhibited relatively high CMA activity and marked heterogeneity at both the single-cell and spatial levels, Myeloid cells were further extracted for re-clustering analysis. Briefly, myeloid cells were normalized, highly variable genes were identified, the data were scaled, and PCA was performed. Harmony was then used for batch correction according to sample origin (22). Based on the ElbowPlot, the first 30 dimensions (dims = 1:30) were retained for downstream analysis. Clustering was initially performed at multiple resolution settings (0.3, 0.5, 0.8, and 1.0), and the final clustering result at resolution = 0.3 was selected based on cluster hierarchy, marker-gene interpretability, and biological plausibility. Cluster relationships across different resolutions were evaluated using clustree, and subtype annotation was performed according to canonical marker genes. In addition, clusters showing likely phagocytosis-related or doublet-like features were excluded before final annotation. UMAP visualization was then performed in the corrected low-dimensional space, and the final myeloid subpopulations were defined based on the selected clustering resolution together with known marker-gene expression patterns.

### 2.5 Differential expression analysis and weighted gene co-expression network analysis

To identify CMA-related candidate genes associated with LUAD development and progression, differential expression analysis between tumor and normal tissues was first performed in the TCGA-LUAD cohort. Subsequently, Single-sample gene set enrichment analysis (ssGSEA) was applied to calculate a CMA score for each sample, and weighted gene co-expression network analysis (WGCNA) was performed in the TCGA-LUAD bulk transcriptomic dataset (23). The soft-thresholding power was evaluated using pickSoftThreshold, and a soft-thresholding power of β = 4 was ultimately selected, at which the scale-free topology fit index (signed R²) reached 0.923 and the mean connectivity was 6.83. This power was chosen as the lowest value satisfying the scale-free topology criterion while preserving sufficient network connectivity and was therefore used to construct the co-expression network and identify the core module most strongly associated with the CMA score. Genes from the core module were then intersected with tumor vs normal differentially expressed genes to obtain candidate genes for downstream modeling.

### 2.6 Functional enrichment analysis

Gene Ontology (GO) enrichment analysis was performed on the candidate genes to evaluate their potential biological functions. Enrichment results were interpreted at the levels of Biological Process, Cellular Component, and Molecular Function, with particular attention to phagocytosis, myeloid immune activation, lipid transport, cell-surface immune interaction, and innate immune recognition-related processes.

### 2.7 Construction and validation of the prognostic signature

To construct a robust CMA-related prognostic model, multiple machine learning algorithms and their combinations were systematically compared based on the candidate genes, including random survival forest (RSF), glmnet, plsRcox, superpc, gbm, CoxBoost, and survivalsvm. Batch-corrected and standardized data from the TCGA-LUAD, GSE31210, and GSE50081 cohorts were used for model training and validation. Predictive performance of different algorithms in the training and validation sets was evaluated using the C-index, and cross-cohort average performance and stability were used as the criteria for final model selection.

Ultimately, the RSF model exhibited the best and most stable predictive performance across cohorts and was therefore selected as the final prognostic model. Based on RSF variable importance, 15 key genes were identified: MGP, CTSH, PDLIM7, METTL7A, CPED1, PLAU, FHL1, ZMYND15, NDRG2, TNFAIP3, G0S2, SLC24A4, ARRDC4, CD1E, and TXNIP. A risk signature was constructed using these genes, and its prognostic stratification ability and generalizability were evaluated in the training and external validation cohorts.

### 2.8 Internal validation by bootstrap optimism correction

To quantify and correct for potential overfitting, we performed internal validation using Harrell’s bootstrap optimism-correction procedure (24, 25), in line with TRIPOD recommendations (26). B = 1,000 bootstrap samples of size n were drawn with replacement from the TCGA training cohort, and the full RSF pipeline (with identical hyperparameters to the final model) was refit on each replicate. The optimism-corrected estimate was obtained as.


$${\hat{\theta }}_{\text{corrected}}={\hat{\theta }}_{\text{apparent}}-\frac{1}{B}\sum_{b=1}^{B}\;({\hat{\theta }}_{\text{train}}^{\left(b\right)}{\hat{\theta }}_{\text{test}}^{\left(b\right)}),$$

where ${\hat{\theta }}_{\text{train}}^{\left(b\right)}$ and ${\hat{\theta }}_{\text{test}}^{\left(b\right)}$ denote performance on the bootstrap sample and on the original cohort, respectively. This was applied to Harrell’s C-index and time-dependent AUCs at 1, 3, and 5 years. Two-sided 95% percentile confidence intervals were derived from the empirical distribution of out-of-sample estimates.

### 2.9 Survival, clinicopathological, and pathway analyses

Patients were divided into high-risk and low-risk groups according to the risk score. Kaplan-Meier analysis was used to compare overall survival between groups, and hazard ratios with 95% confidence intervals were calculated. Time-dependent Receiver operating characteristic (ROC) curves were used to evaluate the predictive performance of the model for 1-year, 3-year, and 5-year overall survival. Univariate and multivariate Cox regression analyses were further performed to assess whether the risk score served as an independent prognostic factor.

In addition, differences in clinical variables, including age, sex, T stage, N stage, M stage, and overall clinical stage, were compared between risk groups, and the distribution of risk scores across clinical subgroups was examined. To investigate the biological differences underlying the two risk groups, GSVA was applied to evaluate hallmark pathway activity, and the correlations between risk score and representative hallmark pathways, together with their prognostic implications, were further analyzed (27).

### 2.10 Immune infiltration and cell-cell communication analyses

To clarify the relationship between the risk signature and the tumor immune microenvironment, multiple immune analysis strategies were applied. ESTIMATE was first used to calculate Stromal Score, Immune Score, and ESTIMATE Score for each sample (28). CIBERSORT was then used to estimate the proportions of 22 immune cell types and to compare infiltration differences between risk groups (29). In addition, ssGSEA was used to evaluate the activity of multiple immune cell subsets and immune-related functional pathways, and Spearman correlations between the 15 signature genes or RiskScore and immune cell infiltration were analyzed.

To explore differences in interactions between epithelial cells and the microenvironment, CellChat was applied to single-cell data (30). Epithelial cells were first divided into high-risk and low-risk groups according to single-cell risk prediction results, and communication networks were then constructed together with other major cell populations. Using CellChatDB. human as the ligand-receptor database, highly expressed genes were identified, interactions were inferred, communication probabilities were calculated, pathway-level signaling was analyzed, and signaling networks were aggregated. Signal directionality was further analyzed for differentially enriched communication patterns, with particular attention to the THBS pathway between fibroblasts and high-risk epithelial cells.

### 2.11 Single-cell validation and drug sensitivity analysis

To validate signature gene expression patterns at the single-cell level, the 15 signature genes were extracted from the single-cell expression matrix and compared across AIS, MIA, and IAC. Pairwise Wilcoxon rank-sum tests were used to compare expression differences among pathological groups, and risk cell proportion plots, dot plots of signature gene expression across cell types, and correlation plots of key genes were generated.

In addition, to assess potential therapeutic response differences between risk groups, the sensitivity of multiple representative anticancer drugs was predicted and estimated IC50 values were compared between the high-risk and low-risk groups (31). The selected drugs included mTOR inhibitors, AKT inhibitors, multi-target kinase inhibitors, apoptosis-regulating agents, epigenetic modulators, as well as classical chemotherapeutic agents, antimetabolites, MEK inhibitors, and PARP inhibitors, in order to evaluate potential treatment vulnerabilities associated with different risk states.

### 2.12 External experimental validation

To further validate the functional role of the core model gene MGP, *in vitro* experiments were performed. An MGP overexpression model was established in the LUAD cell line A549 by plasmid transfection, using 5 μg of overexpression vector per 6-cm dish, and successful overexpression was confirmed at the protein level by Western blot before subsequent functional assays.

For functional assays, CCK-8 assays were first used to evaluate cell proliferation. Transfected cells were seeded into 96-well plates at an appropriate density, absorbance values were measured at designated time points, and proliferation curves were generated to compare the OE-NC and OE-MGP groups. Colony formation assays were then used to assess long-term clonogenic ability. Cells were seeded at low density, cultured until visible colonies formed, and then fixed, stained, and counted to evaluate the effect of MGP on sustained proliferation and colony expansion.

To assess the effect of MGP on migration and invasion, Transwell migration/invasion assays were further performed. For migration assays, treated cells were placed into the upper chamber of Transwell inserts, incubated for a defined period, and then fixed, stained, and quantified based on the number of migrated cells. For invasion assays, the membrane surface was pre-coated with matrix gel to simulate extracellular matrix barriers. The number of cells passing through the membrane was compared between groups to evaluate the effect of MGP on motility and invasive phenotype.

In addition, wound-healing assays were performed to assess lateral migration. Cells were cultured to an appropriate confluence, a linear wound was generated using a sterile pipette tip, and the wound area was recorded at 0 h and 24 h. Wound closure was compared between groups to evaluate the effect of MGP overexpression on migration. Overall, these experiments were used to validate the tumor-suppressive role of MGP in LUAD at the functional level. All experiments were performed with three independent biological replicates, and statistical comparisons between the OE-NC and OE-MGP groups were conducted using a two-tailed unpaired Student’s t-test.

### 2.13 Statistical analysis

All statistical analyses were performed using R software (version 4.5.1). Continuous variables between two groups were compared mainly using the Wilcoxon rank-sum test, and comparisons among multiple groups were performed using appropriate non-parametric tests according to the specific analytical setting. Survival analyses were conducted using the Kaplan-Meier method and Cox proportional hazards regression models. Correlation analyses were performed using Spearman correlation. For analyses involving multiple parallel comparisons, false discovery rate (FDR) or Benjamini-Hochberg (BH) correction was applied where appropriate, including differential expression analyses, functional enrichment analyses, and part of the single-cell and immune-related comparisons. For exploratory analyses, such as some immune infiltration comparisons and drug sensitivity comparisons, original two-sided P values were reported. Unless otherwise specified, all statistical tests were two-sided, and P < 0.05 was considered statistically significant.

## 3 Results

**Figure 1:**
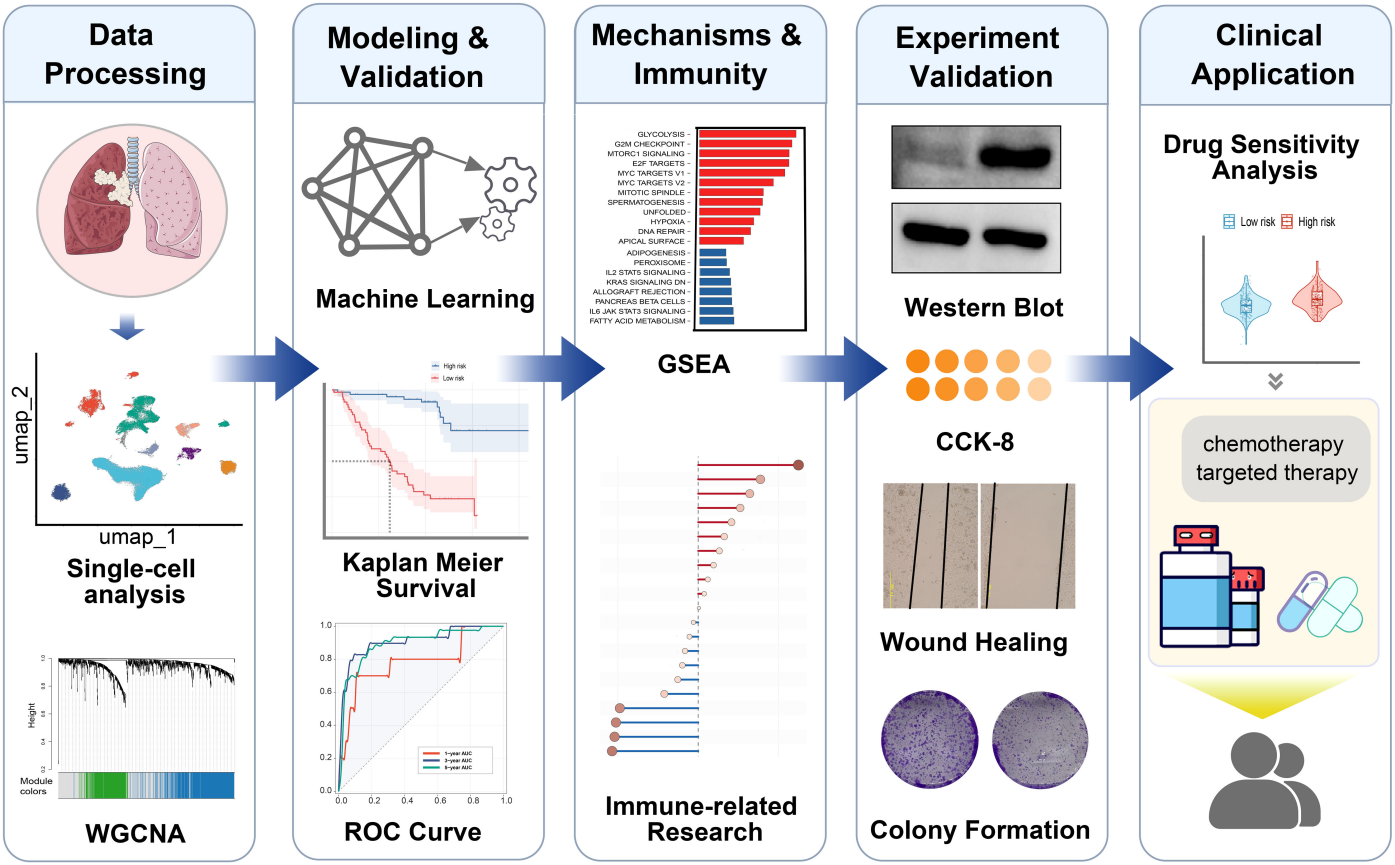
Overview of the study design and analytical workflow.

The overall study design and analytical workflow are summarized in **Figure 1**.

### 3.1 Single-cell landscape reveals cell-type-specific CMA heterogeneity during LUAD progression

**Figure 2:**
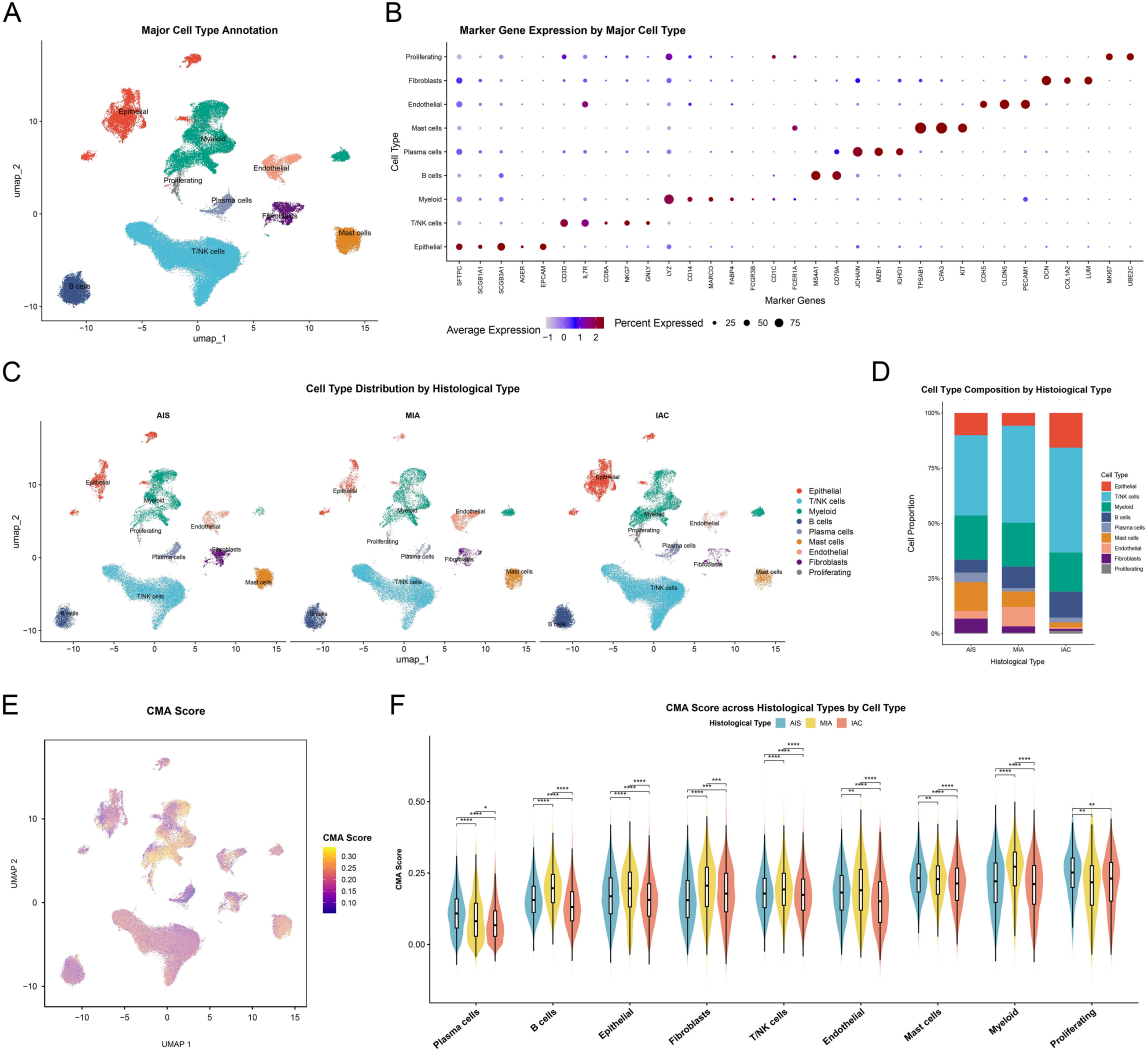
Single-cell landscape and CMA heterogeneity across histological stages in LUAD. **(A)** UMAP plot showing the major cell type annotation of the scRNA-seq dataset. **(B)** Dot plot showing representative marker genes across major cell types. **(C)** UMAP plots showing the distribution of major cell types in AIS, MIA, and IAC samples. **(D)** Stacked bar plot showing cell type composition across histological stages. **(E)** UMAP plot showing the distribution of CMA scores at the single-cell level. **(F)** Violin plots showing CMA score differences across histological stages in each major cell type. ns, not significant; *P < 0.05; **P < 0.01; ***P < 0.001; ****P < 0.0001.

To characterize the cellular composition and CMA activity changes during the AIS/MIA-to-IAC progression of LUAD, we first integrated the GSE189357 single-cell transcriptomic dataset. UMAP visualization showed that all cells could be clearly classified into nine major cell populations, including Epithelial, T/NK cells, Myeloid, B cells, Plasma cells, Mast cells, Endothelial, Fibroblasts, and Proliferating cells (**Figure 2A**). Among these, T/NK cells constituted the dominant population and showed the broadest distribution, whereas Myeloid and Epithelial cells also accounted for a substantial proportion. Plasma cells and Proliferating cells were relatively less abundant. Marker gene analysis further confirmed the reliability of cell annotation. For example, Epithelial cells highly expressed SFTPC, SCGB3A1, and EPCAM, T/NK cells highly expressed CD3D, IL7R, and NKG7, and Myeloid cells highly expressed LYZ, CD14, and MARCO (**Figure 2B**).

We next examined cell distributions across histological stages. AIS, MIA, and IAC samples all contained the major cell populations described above, but their compositions differed markedly among stages (**Figure 2C**). Stacked bar plots showed that T/NK cells were the dominant population across all three stages and tended to increase from AIS to IAC. Epithelial cells accounted for a relatively higher proportion in IAC, whereas Endothelial cells were more prominent in MIA. In contrast, Mast cells and Plasma cells were relatively less abundant in IAC (**Figure 2D**). These findings suggest that progression from AIS to IAC is accompanied not only by changes in the tumor epithelial compartment, but also by concurrent remodeling of immune and stromal components.

At the single-cell level, CMA activity displayed a non-uniform distribution across the UMAP space, indicating pronounced cell type specificity rather than homogeneous expression across all cells (**Figure 2E**). Comparison of CMA scores across histological stages within each cell type revealed significant stage-specific differences in nearly all major cell populations. Specifically, B cells, Epithelial cells, Fibroblasts, T/NK cells, Endothelial cells, and Myeloid cells generally exhibited higher CMA scores in MIA, whereas partial declines were observed in some cell types in IAC (**Figure 2F**). Among them, Myeloid cells showed consistently high CMA scores overall and exhibited one of the most prominent stage-dependent changes. These results indicate that CMA-related transcriptional programs are strongly cell type-dependent during the AIS/MIA-to-IAC progression of LUAD, particularly in immune and tumor-associated cell populations, thereby providing a basis for subsequent focus on the myeloid compartment and construction of a CMA-related prognostic model.

### 3.2 Spatial mapping and myeloid re-clustering reveal dynamic remodeling of the myeloid compartment

**Figure 3:**
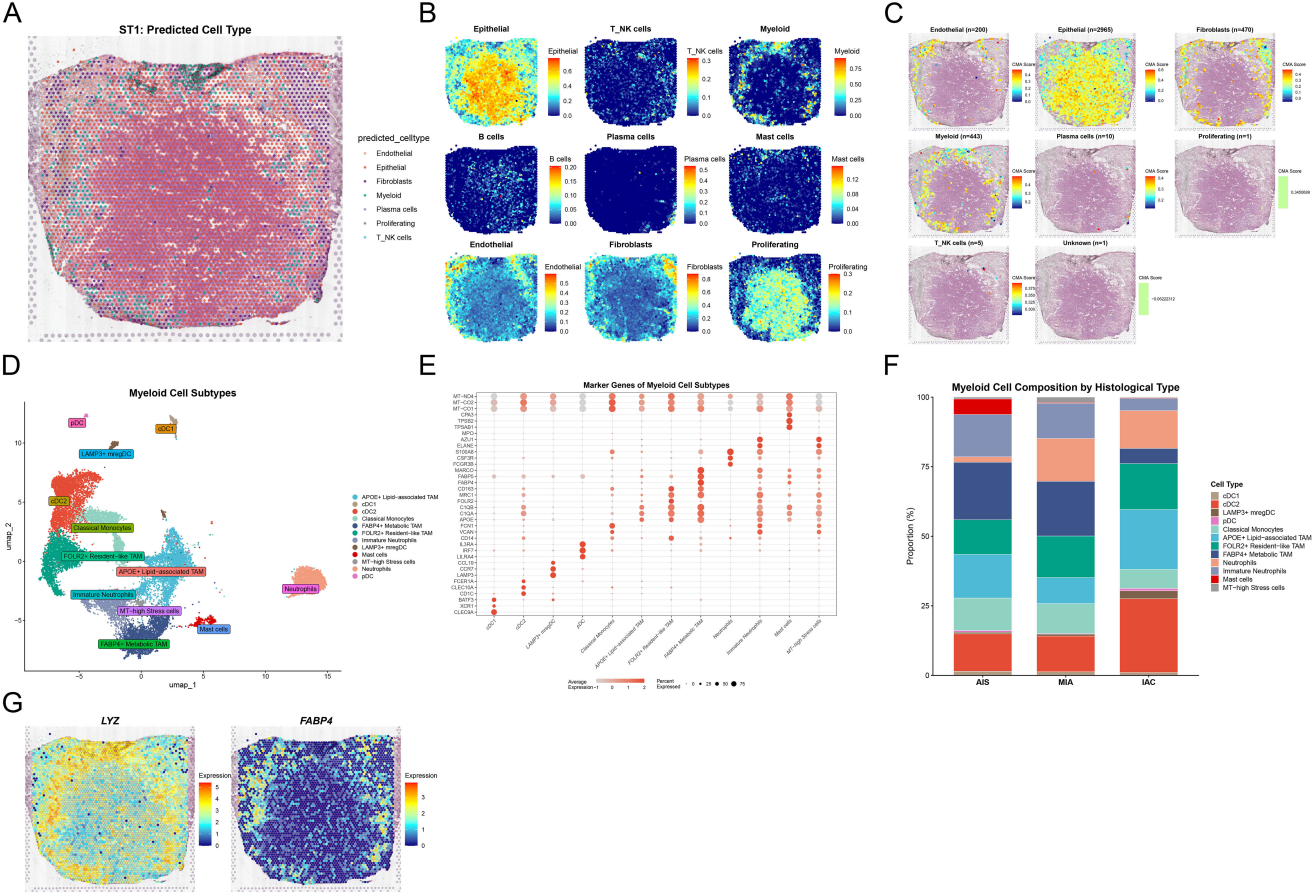
Spatial mapping and myeloid re-clustering analysis in LUAD. **(A)** Spatial distribution of predicted major cell types in the ST1 section. **(B)** Spatial feature plots showing the enrichment patterns of major cell types. **(C)** Spatial distribution of CMA scores across different cell types. **(D)** UMAP plot showing the re-clustered myeloid cell subtypes. **(E)** Dot plot showing representative marker genes across myeloid cell subtypes. **(F)** Stacked bar plot showing the composition of myeloid cell subtypes across AIS, MIA, and IAC. **(G)** Spatial feature plots showing the expression patterns of LYZ and FABP4.

To further characterize the spatial distribution of different cell populations in LUAD tissues and clarify myeloid heterogeneity during pathological progression, we integrated spatial transcriptomic data for cell type mapping and myeloid re-clustering analysis. Spatial localization analysis showed marked spatial heterogeneity of different cell types within the first spatial transcriptomic section, with Epithelial cells occupying most regions and constituting the dominant tissue component, whereas Endothelial cells, Fibroblasts, Myeloid cells, and other immune populations were distributed in more scattered or focal patterns (**Figure 3A**).

Further examination of spatial enrichment patterns revealed non-uniform distribution of the major cell populations. Epithelial signals were broadly enriched in large contiguous regions, indicating spatial dominance of tumor epithelial components. Myeloid signals were mainly scattered but showed relatively concentrated enrichment in certain focal areas. Fibroblasts and Endothelial cells displayed more localized regional distributions, reflecting the focal nature of stromal components. By contrast, B cells, Plasma cells, Mast cells, and Proliferating cells showed overall weak signals and were detected only at low levels in limited local regions (**Figure 3B**).

Spatial CMA scores also showed clear heterogeneity. Epithelial, Myeloid, Fibroblast, and Endothelial cells contained more regions with high CMA scores, whereas Plasma cells, T/NK cells, and Proliferating cells were represented by fewer spots and showed weaker overall spatial signals. Notably, Epithelial cells were not only the most abundant but also exhibited widespread high-CMA regions, while Myeloid cells, although less abundant than Epithelial cells, still displayed clear focal enrichment of high CMA signals (**Figure 3C**). These findings suggest that CMA-related activity is characterized not only by cell type specificity but also by distinct spatial organization.

Given the prominent CMA-related features observed in myeloid cells at both the single-cell and spatial levels, the Myeloid compartment was further re-clustered. UMAP analysis identified multiple transcriptionally distinct myeloid subpopulations, including cDC1, cDC2, LAMP3+ mregDC, pDC, Classical Monocytes, APOE+ Lipid-associated TAM, FOLR2+ Resident-like TAM, FABP4+ Metabolic TAM, Immature Neutrophils, Mature Neutrophils, MT-high Stress cells, and Mast cells (**Figure 3D**). Among them, cDC2, FOLR2+ Resident-like TAM, FABP4+ Metabolic TAM, and APOE+ Lipid-associated TAM constituted relatively large cell populations, whereas cDC1, pDC, LAMP3+ mregDC, and Mast cells were relatively scarce (**Figure 3D**).

Marker gene expression patterns further supported these annotations. The dot plot showed that dendritic cell-related subsets expressed canonical antigen presentation-associated genes, whereas distinct TAM subsets exhibited marker profiles associated with lipid metabolism, tissue residency, or metabolic reprogramming. Neutrophil-related subsets showed the expected granulocytic marker signatures (**Figure 3E**). This clear molecular stratification indicates that myeloid cells in LUAD do not represent a single homogeneous population, but rather a collection of functionally distinct states.

The composition of myeloid subsets across histological stages further revealed marked state remodeling. Compared with AIS and MIA, cDC2 showed the highest proportion in IAC, and APOE+ Lipid-associated TAM was also clearly enriched in IAC. In contrast, FABP4+ Metabolic TAM was mainly distributed in AIS and MIA and was markedly reduced in IAC. At the same time, Immature Neutrophils showed the highest proportion in AIS and gradually declined during progression, reaching the lowest level in IAC (**Figure 3F**). By comparison, FOLR2+ Resident-like TAM remained relatively stable across the three stages, although slightly increased in IAC. These results suggest that progression from early lesions to invasive adenocarcinoma is not characterized by simple expansion of the myeloid compartment, but rather by dynamic remodeling from early inflammatory-like and metabolic states toward dendritic-like and lipid-associated macrophage states.

Finally, representative myeloid-related marker genes were visualized in the spatial transcriptomic sections. LYZ was broadly distributed throughout the tissue and maintained relatively high expression over a large area, whereas FABP4 exhibited a more focal distribution and was mainly concentrated in selected local regions (**Figure 3G**). This finding is consistent with the presence of distinct TAM states identified in the myeloid subset analysis and further supports pronounced heterogeneity in the composition and functional states of myeloid cells in LUAD tissues.

### 3.3 WGCNA identifies CMA-related candidate genes in LUAD

**Figure 4:**
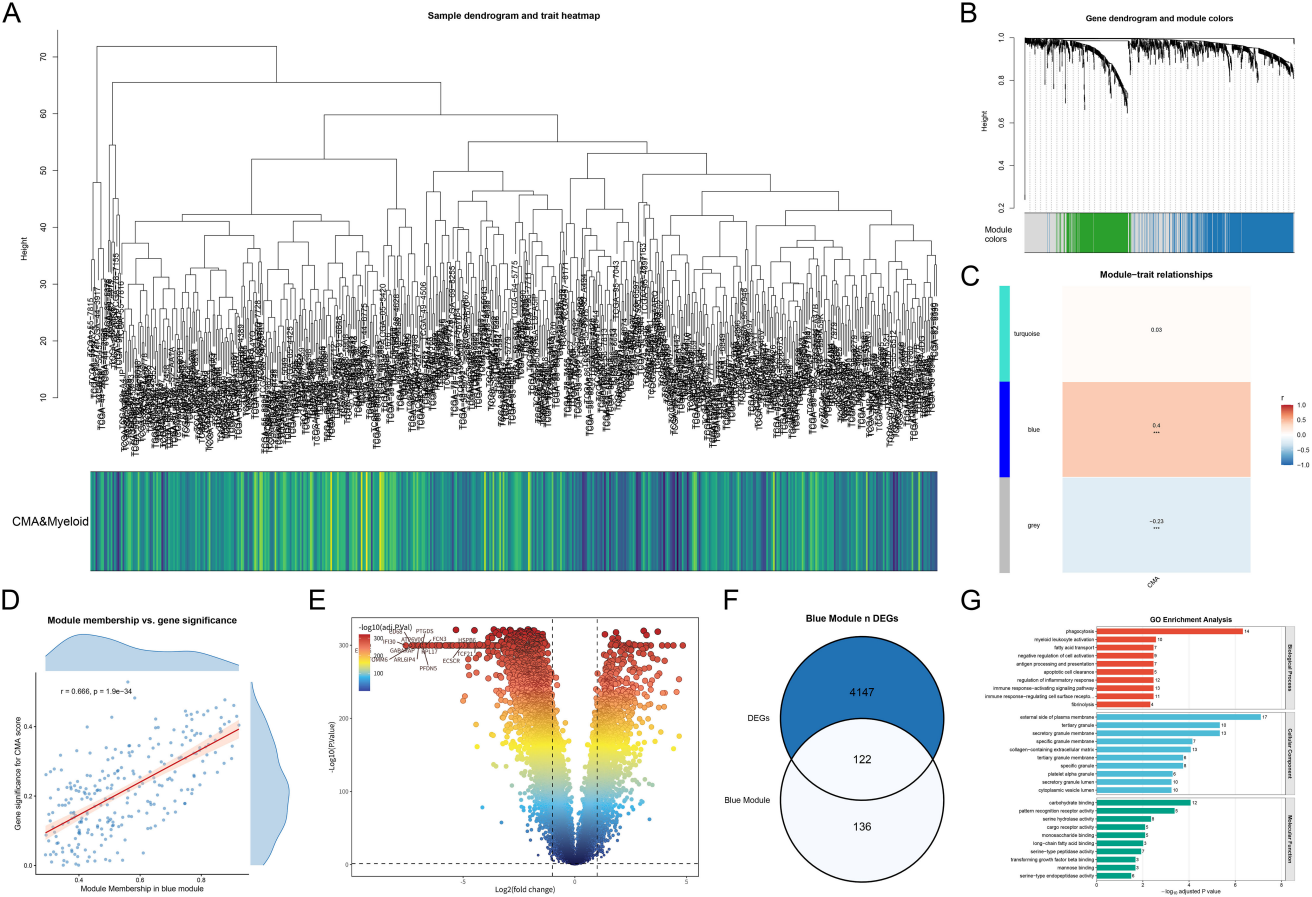
Identification of CMA-related candidate genes by WGCNA and differential expression analysis. **(A)** Sample clustering dendrogram with the CMA trait heatmap. **(B)** Gene dendrogram showing module assignment. **(C)** Heatmap showing correlations between gene modules and CMA score. **(D)** Scatter plot showing the correlation between module membership and gene significance in the blue module. **(E)** Volcano plot of differentially expressed genes between tumor and normal samples in the TCGA-LUAD cohort. **(F)** Venn diagram showing the overlap between blue module genes and tumor-normal differentially expressed genes. **(G)** GO enrichment analysis of the intersected candidate genes. *P < 0.05; **P < 0.01; ***P < 0.001; ****P < 0.0001.

To further identify key genes closely associated with CMA activity at the bulk transcriptomic level, we performed weighted gene co-expression network analysis using the TCGA-LUAD cohort. A soft-thresholding power of β = 4 was selected for network construction, at which the scale-free topology fit index (signed R²) reached 0.923 and the mean connectivity was 6.83 (**Supplementary Figure 1**). The sample clustering dendrogram showed that the samples were overall stably clustered without obvious outliers, indicating reliable data quality suitable for subsequent network construction (**Figure 4A**). Hierarchical clustering of genes further divided the candidate genes into multiple co-expression modules, among which the turquoise and blue modules were the major non-grey modules (**Figure 4B**).

We next assessed the correlations between these modules and the CMA phenotype. The module-trait relationship heatmap showed that the blue module was most positively correlated with the CMA score, whereas the grey module was negatively correlated with the CMA score, and the turquoise module showed a relatively weaker association (**Figure 4C**). The blue module showed a correlation coefficient of 0.40 and reached statistical significance and was therefore selected as the core module for subsequent analysis. Further analysis of the relationship between module membership and gene significance within the blue module showed a strong positive correlation (r = 0.666, P < 0.0001), indicating that highly connected genes within this module also tended to show stronger associations with CMA (**Figure 4D**).

Meanwhile, differential expression analysis between tumor and normal tissues in the TCGA-LUAD cohort revealed a large number of significantly altered genes, indicating widespread transcriptional dysregulation at the bulk level in LUAD (**Figure 4E**). Intersecting these tumor vs normal differentially expressed genes with the blue module genes yielded 122 candidate genes, which were used as input features for subsequent machine learning modeling (**Figure 4F**). This screening strategy established a link between CMA-related co-expression patterns and LUAD-associated differential transcriptional features, thereby improving the disease relevance of the candidate genes.

GO enrichment analysis of these 122 candidate genes showed that they were mainly involved in myeloid immune activation, phagocytosis, and metabolic remodeling-related functions. At the Biological Process level, the candidate genes were significantly enriched in phagocytosis, myeloid leukocyte activation, and fatty acid transport, among which phagocytosis showed the highest significance and involved the largest number of candidate genes. At the Cellular Component level, these genes were mainly localized to the external side of plasma membrane, tertiary granule, and specific granule membrane. At the Molecular Function level, they were mainly enriched in carbohydrate binding and pattern recognition receptor activity (**Figure 4G**). These results indicate that the CMA-related candidate genes identified here are closely associated with myeloid cell phagocytic activation, lipid transport, cell-surface immune interactions, and innate immune recognition.

### 3.4 Machine learning establishes and validates a robust CMA-related prognostic signature

**Figure 5:**
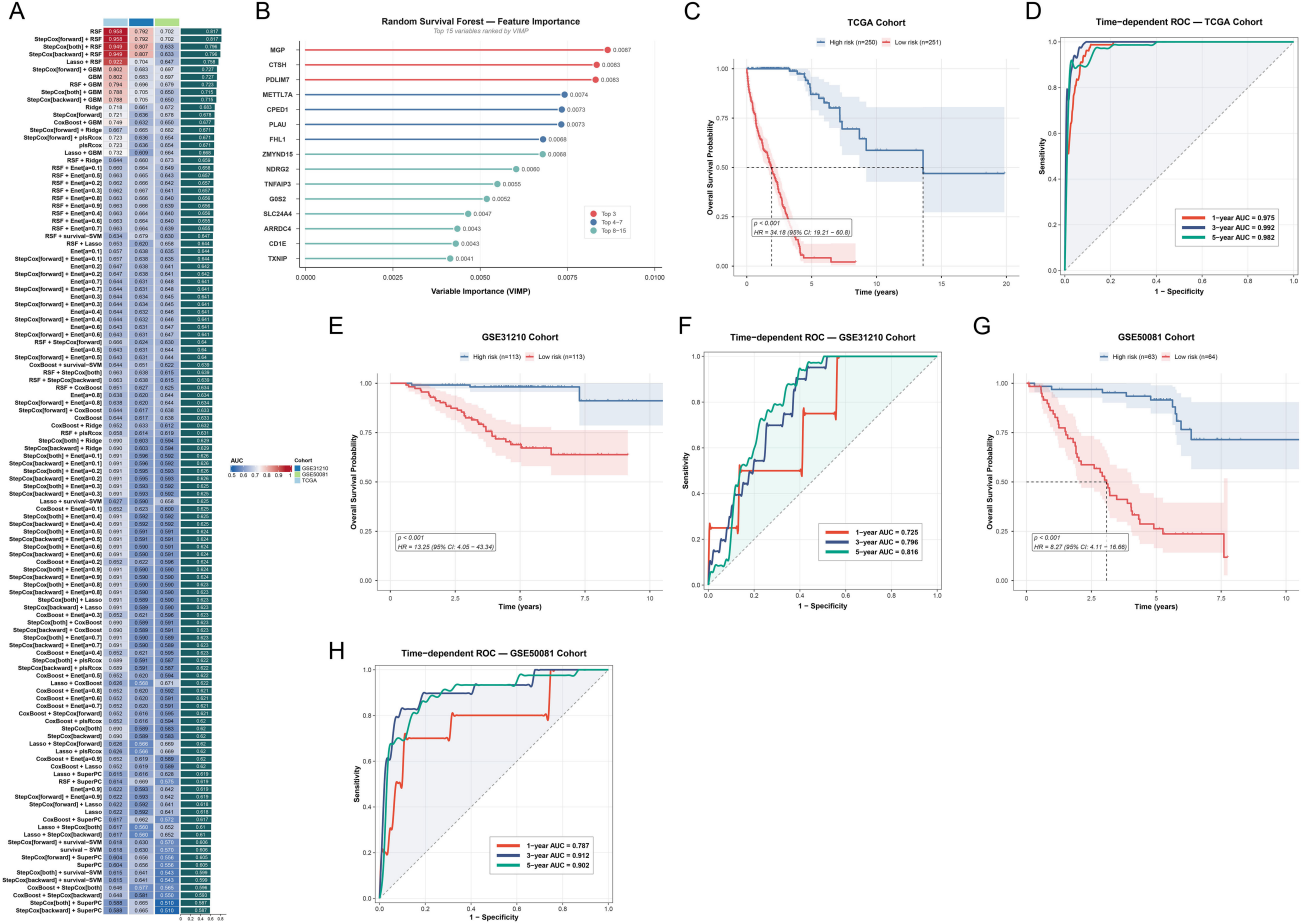
Construction and validation of the CMA-related prognostic signature using machine learning. **(A)** Heatmap showing the C-index of different machine learning algorithms across the TCGA, GSE31210, and GSE50081 cohorts. **(B)** Variable importance plot of the top 15 genes selected by the RSF model. **(C)** Kaplan-Meier survival curves for the high-risk and low-risk groups in the TCGA cohort. **(D)** Time-dependent ROC curves for 1-, 3-, and 5-year overall survival in the TCGA cohort. **(E)** Kaplan-Meier survival curves for the high-risk and low-risk groups in the GSE31210 cohort. **(F)** Time-dependent ROC curves for 1-, 3-, and 5-year overall survival in the GSE31210 cohort. **(G)** Kaplan-Meier survival curves for the high-risk and low-risk groups in the GSE50081 cohort. **(H)** Time-dependent ROC curves for 1-, 3-, and 5-year overall survival in the GSE50081 cohort. *P < 0.05, **P < 0.01, ***P < 0.001, ****P < 0.0001.

To construct a robust CMA-related prognostic model, we systematically compared the predictive performance of multiple machine learning algorithms and their combinations based on the 122 candidate genes identified above. The integrated heatmap showed marked differences in C-index performance across the TCGA, GSE31210, and GSE50081 cohorts, among which models centered on RSF performed best overall. In particular, RSF alone, as well as its combinations with StepCox[forward], StepCox[both], and StepCox[backward], ranked among the top-performing models, suggesting that random survival forest had strong generalizability and stability in this analytical framework (**Figure 5A**). Notably, the standalone RSF model achieved C-index values of 0.958, 0.792, and 0.702 in the TCGA, GSE31210, and GSE50081 cohorts, respectively, with an average C-index of 0.817, which was the highest among all candidate models. Therefore, it was selected as the final modeling method (**Figure 5A**).

Based on the variable importance output of the final RSF model, 15 genes with the greatest contribution to prognostic stratification were identified: MGP, CTSH, PDLIM7, METTL7A, CPED1, PLAU, FHL1, ZMYND15, NDRG2, TNFAIP3, G0S2, SLC24A4, ARRDC4, CD1E, and TXNIP. Among them, MGP had the highest importance score, followed by CTSH and PDLIM7, whereas CD1E and TXNIP had relatively lower importance but were still retained in the final signature (**Figure 5B**). This result indicates that the final model does not rely on a single biomarker, but rather integrates multiple key genes associated with CMA-related and myeloid-related biological processes.

A risk model was then constructed based on these 15 genes, and its prognostic stratification ability was evaluated in the training set and two external validation cohorts. In the TCGA cohort, patients in the high-risk group had significantly worse overall survival than those in the low-risk group. Kaplan-Meier curves separated early and remained clearly distinct throughout follow-up. The high-risk group had a markedly shortened median survival, with an HR of 34.18 (95% CI: 19.21–60.8, P < 0.001), indicating extremely strong discriminatory power for survival (**Figure 5C**). Time-dependent ROC analysis further showed that the model achieved 1-year, 3-year, and 5-year Area under the curve (AUC) values of 0.975, 0.992, and 0.982, respectively, in the TCGA cohort, all at exceptionally high levels, indicating excellent short-term and mid-term prognostic performance (**Figure 5D**).

In the external validation cohorts, this signature likewise showed stable prognostic stratification ability. In GSE31210, the survival probability of the high-risk group remained consistently lower than that of the low-risk group, with progressively increasing separation during follow-up. The HR was 13.25 (95% CI: 4.05–43.34, P < 0.001) (**Figure 5E**). The corresponding time-dependent ROC curves showed 1-year, 3-year, and 5-year AUC values of 0.725, 0.796, and 0.816, respectively, indicating that the model retained good predictive accuracy in this validation cohort (**Figure 5F**). In GSE50081, Kaplan-Meier curves likewise showed significantly poorer prognosis in the high-risk group, with an HR of 8.27 (95% CI: 4.11–16.66, P < 0.001), and the separation between the two groups became even more pronounced after approximately 3 years of follow-up (**Figure 5G**). In addition, the model achieved 1-year, 3-year, and 5-year AUC values of 0.787, 0.912, and 0.902, respectively, in GSE50081, further supporting its cross-cohort robustness and generalizability (**Figure 5H**).

Overall, the 15-gene CMA-related signature established by RSF stably distinguished patients with different prognostic risks in the training set and two independent validation cohorts, with high predictive accuracy, highlighting its potential clinical utility.

### 3.5 Internal validation confirms robust discrimination

Bootstrap optimism correction (B = 1,000) yielded moderate optimism (0.08–0.10) across all metrics. After correction, the model retained strong discrimination, with a C-index of 0.856 (95% CI 0.840–0.885) and time-dependent AUCs of 0.884, 0.913, and 0.901 at 1, 3, and 5 years (**Table 1**). The corrected C-index closely matched that of the two independent GEO cohorts (mean ≈ 0.82), supporting genuine generalizability rather than overfitting.

**Table 1:** Bootstrap optimism-corrected internal validation (B = 1,000).

Metric	Apparent	Optimism	Corrected	95% CI
C-index	0.958	0.103	0.856	0.840–0.885
AUC at 1 year	0.983	0.099	0.884	0.851–0.924
AUC at 3 years	0.996	0.083	0.913	0.889–0.936
AUC at 5 years	0.995	0.094	0.901	0.868–0.929

### 3.6 The risk signature is closely associated with clinicopathological progression

**Figure 6:**
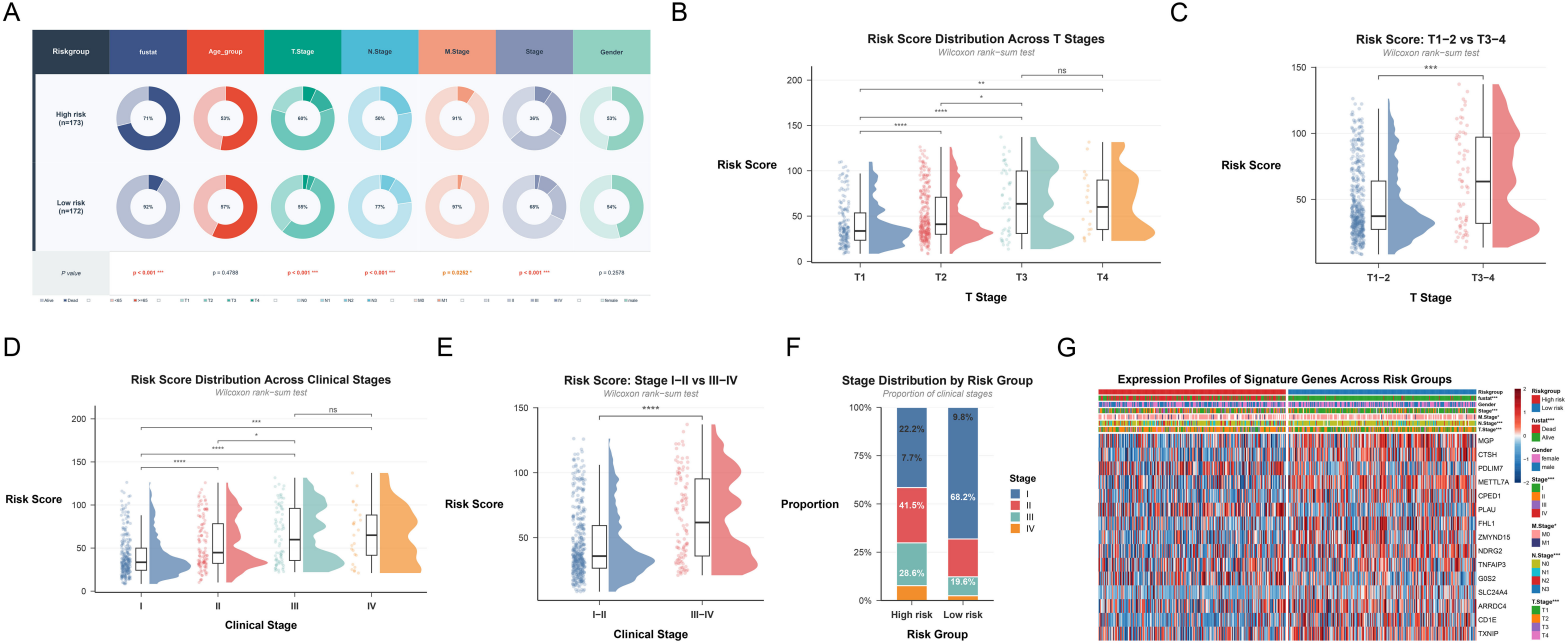
Association of the CMA-related risk signature with clinicopathological characteristics. **(A)** Donut plots showing the distribution of clinical variables between the high-risk and low-risk groups. **(B)** Violin plots showing risk score distribution across different T stages. **(C)** Comparison of risk scores between T1–2 and T3–4 groups. **(D)** Violin plots showing risk score distribution across different clinical stages. **(E)** Comparison of risk scores between stage I–II and stage III–IV groups. **(F)** Stacked bar plot showing clinical stage composition in the high-risk and low-risk groups. **(G)** Heatmap showing the expression profiles of the 15 signature genes across the two risk groups together with clinicopathological annotations. ns, not significant; *P < 0.05, **P < 0.01, ***P < 0.001, ****P < 0.0001.

To further evaluate the relationship between this CMA-related risk model and clinicopathological characteristics, we compared the distribution of multiple clinical variables between the high-risk and low-risk groups. Donut plots showed significant differences between the two groups in fustat, T stage, N stage, M stage, and overall stage, whereas age group and gender were similarly distributed and showed no significant differences (**Figure 6A**). Specifically, the high-risk group had a higher proportion of death events and a higher proportion of patients with advanced T stage, lymph node metastasis, distant metastasis, and advanced clinical stage, indicating that the risk score is closely associated with disease progression.

We next analyzed the distribution of risk scores across different clinical subgroups. Stratified by T stage, the risk score generally increased with increasing T stage, with patients at T3 and T4 showing overall higher scores than those at T1 and T2. Group comparisons demonstrated significant differences between T1 and T2, T1 and T3, T1 and T4, and T2 and T3, whereas no significant difference was observed between T3 and T4, suggesting that the risk score can effectively distinguish early from advanced local tumor burden (**Figure 6B**). When patients were further grouped into T1–2 and T3–4, the advanced T-stage group still showed significantly elevated risk scores, further confirming this trend (**Figure 6C**).

Similarly, in the analysis of overall clinical stage, the risk score also showed an overall increasing trend with advancing stage. Patients with stage III and stage IV disease had higher risk scores than those with stage I and stage II disease. Significant differences were observed between stage I and stage II, stage I and stage III, stage I and stage IV, and stage II and stage III, whereas no significant difference was observed between stage III and stage IV (**Figure 6D**). When patients were further grouped into stage I–II and stage III–IV, those with advanced-stage disease still exhibited significantly higher risk scores than those with early-stage disease (**Figure 6E**). Consistently, the stacked bar plot of stage composition showed that stage III patients were markedly enriched in the high-risk group, whereas stage I patients were more frequent in the low-risk group, further supporting the close relationship between the risk score and clinical progression of LUAD (**Figure 6F**).

Finally, we compared the expression patterns of the 15 signature genes between the two risk groups. The heatmap showed clear separation of gene expression profiles between the high-risk and low-risk groups, accompanied by distinct distributions of survival status and clinical characteristics (**Figure 6G**). Most signature genes showed obvious differential expression between the two groups, indicating that this risk model not only reflects prognostic heterogeneity among patients but also captures molecular features associated with different clinical progression states.

Overall, this CMA-related risk signature was closely associated with T stage, overall clinical stage, and survival outcome in LUAD. The high-risk state generally corresponded to more advanced clinicopathological features and poorer outcomes, underscoring its potential value for clinical stratification.

### 3.7 The risk signature independently predicts prognosis and reflects distinct pathway activity patterns

**Figure 7:**
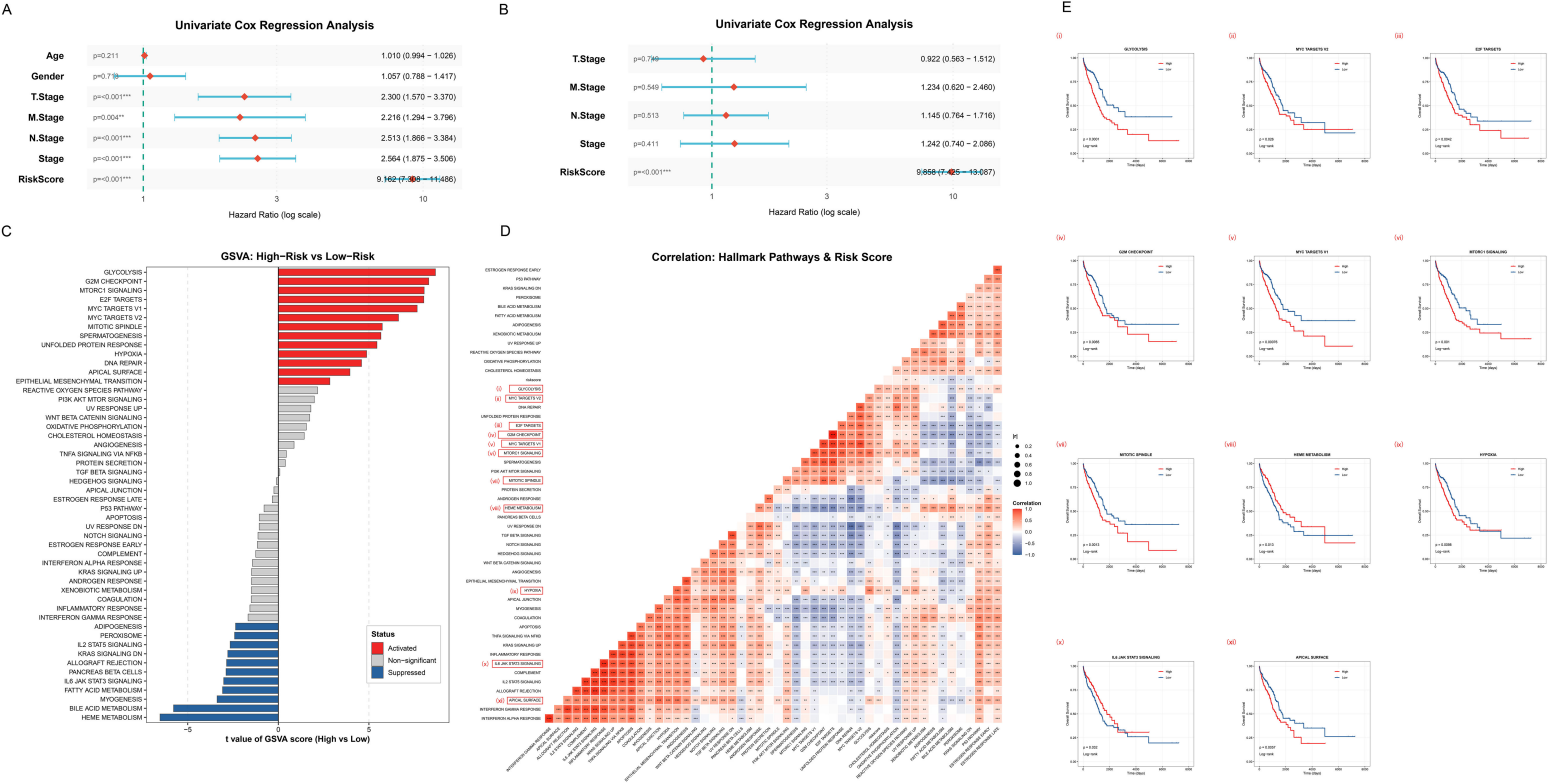
Independent prognostic value and pathway characteristics of the CMA-related risk signature. **(A)** Univariate Cox regression analysis of clinicopathological variables and risk score. **(B)** Multivariate Cox regression analysis of clinicopathological variables and risk score. **(C)** GSVA showing hallmark pathway differences between the high-risk and low-risk groups. **(D)** Correlation heatmap showing the associations between hallmark pathways and risk score. **(E)** Kaplan-Meier survival analyses of representative hallmark pathways. ns, not significant; *P < 0.05, **P < 0.01, ***P < 0.001, ****P < 0.0001.

To further assess the independent prognostic value of this risk signature, Cox regression analyses were first performed. In univariate Cox regression, T stage, M stage, N stage, overall stage, and RiskScore were all significantly associated with overall survival, whereas age and gender were not. Among them, RiskScore showed the highest hazard ratio, indicating the strongest association with patient outcome (**Figure 7A**). After further adjustment for clinical variables in multivariate Cox regression, RiskScore remained significant, whereas the other clinical variables lost statistical significance, indicating that this signature can independently predict the prognosis of patients with LUAD beyond conventional clinicopathological indicators (**Figure 7B**).

To explore the biological differences underlying the two risk groups, GSVA was further performed. The results showed that the high-risk group was significantly enriched in multiple hallmark pathways closely related to malignant tumor progression, including GLYCOLYSIS, G2M CHECKPOINT, MTORC1 SIGNALING, E2F TARGETS, MYC TARGETS V1, MYC TARGETS V2, and MITOTIC SPINDLE. Among these, GLYCOLYSIS showed the most prominent difference, suggesting that high-risk patients may exhibit more active metabolic reprogramming, whereas elevated G2M CHECKPOINT, MTORC1 SIGNALING, and E2F TARGETS suggest stronger cell-cycle progression and proliferative activity in the high-risk group. In contrast, the low-risk group was relatively enriched in HEME METABOLISM, BILE ACID METABOLISM, MYOGENESIS, FATTY ACID METABOLISM, and IL6 JAK STAT3 SIGNALING, suggesting that low-risk patients may retain more metabolism-related homeostatic features (**Figure 7C**).

Further systematic analysis of the correlations between risk score and hallmark pathways revealed that these 11 representative pathways could be clearly categorized into high-risk-associated and low-risk-associated pathways. Specifically, GLYCOLYSIS, MYC TARGETS V2, E2F TARGETS, G2M CHECKPOINT, MYC TARGETS V1, MTORC1 SIGNALING, MITOTIC SPINDLE, HYPOXIA, and APICAL SURFACE were positively correlated with the risk score, indicating that the high-risk state is mainly characterized by glycolytic reprogramming, MYC/E2F-driven proliferative programs, cell-cycle progression, mTORC1 activation, and hypoxia-related features. By contrast, HEME METABOLISM and IL6 JAK STAT3 SIGNALING were negatively correlated with the risk score, indicating that these two pathways were more closely associated with the low-risk state (**Figure 7D**).

To further evaluate the clinical relevance of these representative hallmark pathways, Kaplan-Meier survival analyses were performed for each of the 11 pathways. Among the high-risk-associated pathways, high expression of GLYCOLYSIS, MYC TARGETS V2, E2F TARGETS, G2M CHECKPOINT, MYC TARGETS V1, MTORC1 SIGNALING, MITOTIC SPINDLE, HYPOXIA, and APICAL SURFACE was associated with significantly worse overall survival. In contrast, among the low-risk-associated pathways, high expression of HEME METABOLISM and IL6 JAK STAT3 SIGNALING was consistent with relatively better survival outcomes (**Figure 7E**). These findings indicate that the CMA-related risk signature not only independently predicts prognosis in LUAD, but also effectively reflects core biological pathways closely linked to prognosis across different risk states.

### 3.8 The risk signature is associated with a distinct immune microenvironment

**Figure 8:**
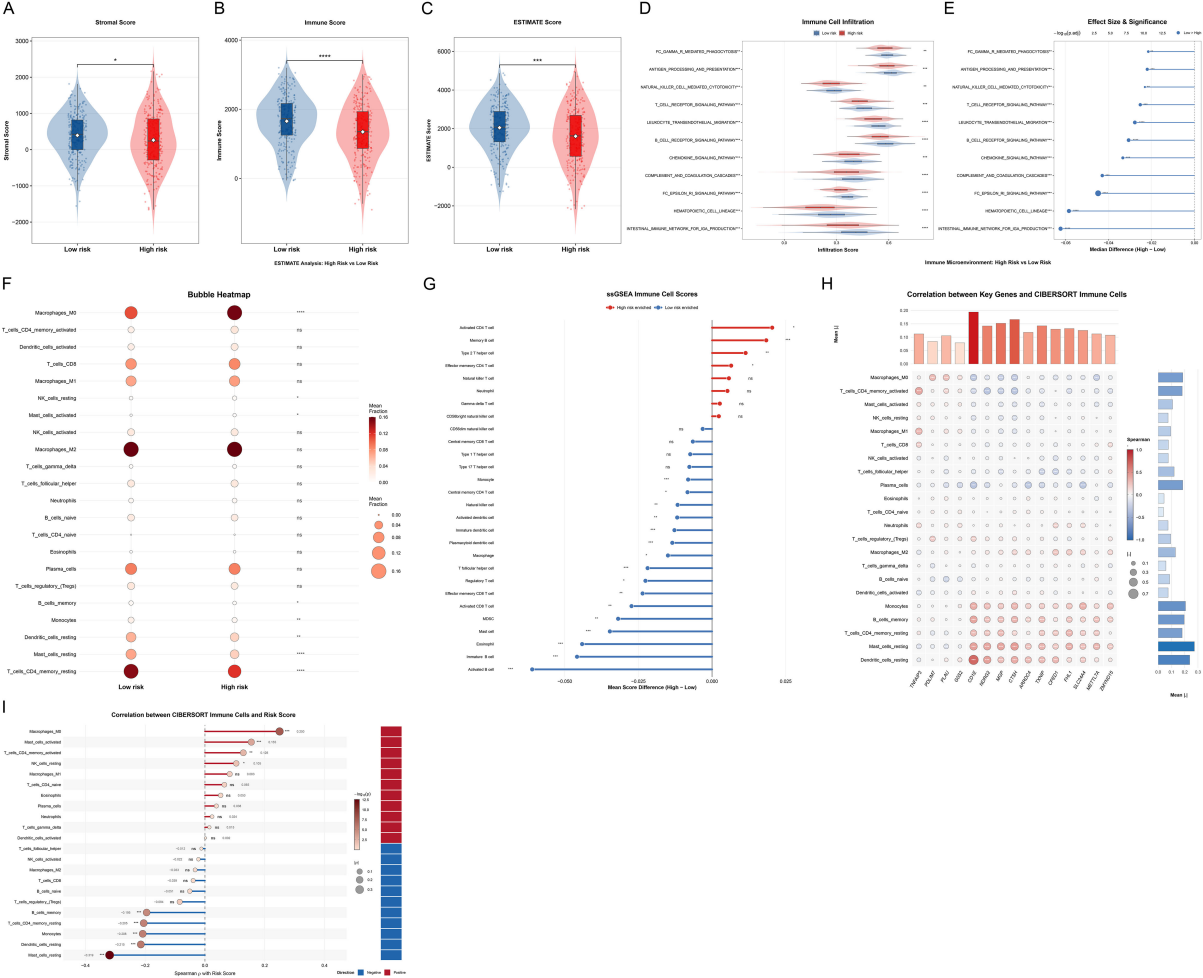
Immune microenvironment characteristics associated with the CMA-related risk signature. **(A)** Comparison of stromal score between the low-risk and high-risk groups. **(B)** Comparison of immune score between the low-risk and high-risk groups. **(C)** Comparison of estimate score between the low-risk and high-risk groups. **(D)** Violin plots showing differences in immune-related pathway activity between the two risk groups. **(E)** Effect size and significance of immune-related pathway differences between the two risk groups. **(F)** Bubble heatmap showing differences in CIBERSORT immune cell fractions between the low-risk and high-risk groups. **(G)** ssGSEA-based comparison of immune cell scores between the two risk groups. **(H)** Correlation heatmap showing the associations between the 15 signature genes and CIBERSORT immune cells. **(I)** Correlation between RiskScore and CIBERSORT immune cell infiltration. ns, not significant; *P < 0.05, **P < 0.01, ***P < 0.001, ****P < 0.0001.

To further clarify the relationship between this CMA-related risk signature and the tumor immune microenvironment, we first used the ESTIMATE algorithm to compare stromal and immune components between the high-risk and low-risk groups. The results showed that Stromal Score, Immune Score, and ESTIMATE Score were all significantly higher in the low-risk group than in the high-risk group, indicating that low-risk patients harbored both greater stromal content and more active immune infiltration, whereas the high-risk group showed relatively lower stromal and immune microenvironment scores overall (**Figures 8A–C**).

We next analyzed differences in immune-related functional pathways between the two risk groups. The results showed that FC gamma R mediated phagocytosis, antigen processing and presentation, natural killer cell mediated cytotoxicity, T cell receptor signaling pathway, leukocyte transendothelial migration, B cell receptor signaling pathway, chemokine signaling pathway, complement and coagulation cascades, FC epsilon RI signaling pathway, hematopoietic cell lineage, and intestinal immune network for IGA production were all overall higher in the low-risk group, indicating that low-risk patients retained stronger antigen presentation, T-cell and B-cell receptor signaling, NK cell-mediated cytotoxicity, and chemokine-related immune responses (**Figures 8D, E**). Overall, the low-risk group exhibited a more complete immune activation and immune response profile, whereas the high-risk group showed a relatively weakened immune functional state.

At the level of immune cell infiltration, CIBERSORT analysis showed that the two risk groups differed in the abundance of multiple immune cell populations. The bubble heatmap indicated that Macrophages M0 were more enriched in the high-risk group, whereas Mast cells resting, Dendritic cells resting, Monocytes, B cells memory, and T cells CD4 memory resting were higher in the low-risk group. In addition, Mast cells activated and NK cells resting also showed a relatively increasing trend in the high-risk group (**Figure 8F**). These results suggest that the high-risk state is characterized by a microenvironment biased toward undifferentiated or non-classically activated myeloid infiltration, whereas the low-risk group retains more resting dendritic cells, monocytes, and memory lymphocyte components.

The immune cell scoring results derived from ssGSEA further supported this pattern. The high-risk group was mainly enriched in Activated CD4 T cell, Memory B cell, Type 2 T helper cell, and Effector memory CD4 T cell, whereas the low-risk group showed more prominent enrichment of Activated B cell, Immature B cell, Eosinophil, Mast cell, MDSC, Activated CD8 T cell, Effector memory CD8 T cell, Regulatory T cell, and T follicular helper cell (**Figure 8G**). Overall, the low-risk group exhibited broader immune cell recruitment and infiltration, whereas the immune enrichment pattern of the high-risk group was relatively limited.

Further analysis of correlations between the 15 signature genes and CIBERSORT immune cells revealed clearly distinct association patterns. Overall, some key genes were strongly correlated with Monocytes, B cells memory, T cells CD4 memory resting, Dendritic cells resting, and Mast cells resting, whereas other genes were more closely associated with Macrophages M0, Mast cells activated, and NK cells resting, suggesting that this signature may shape different immune infiltration patterns through coordinated multi-gene effects (**Figure 8H**).

Finally, we further evaluated the correlations between RiskScore and immune cell infiltration. RiskScore was positively correlated with Macrophages M0, Mast cells activated, and NK cells resting, with the strongest positive correlation observed for Macrophages M0. In contrast, Mast cells resting, Dendritic cells resting, Monocytes, T cells CD4 memory resting, and B cells memory were negatively correlated with RiskScore, indicating that as the risk score increased, the tumor microenvironment progressively shifted toward a state characterized by immature macrophages and activated mast cells, whereas cell populations related to antigen presentation and memory immunity gradually declined (**Figure 8I**).

Taken together, this CMA-related risk signature was closely associated with immune microenvironment remodeling in LUAD. The low-risk group exhibited higher Stromal Score, Immune Score, and ESTIMATE Score, together with more active immune functions, whereas the high-risk group showed globally weakened immune activation and enrichment of specific myeloid and mast cell-related components.

### 3.9 Single-cell validation highlights THBS1 signaling in high-risk epithelial cells

**Figure 9:**
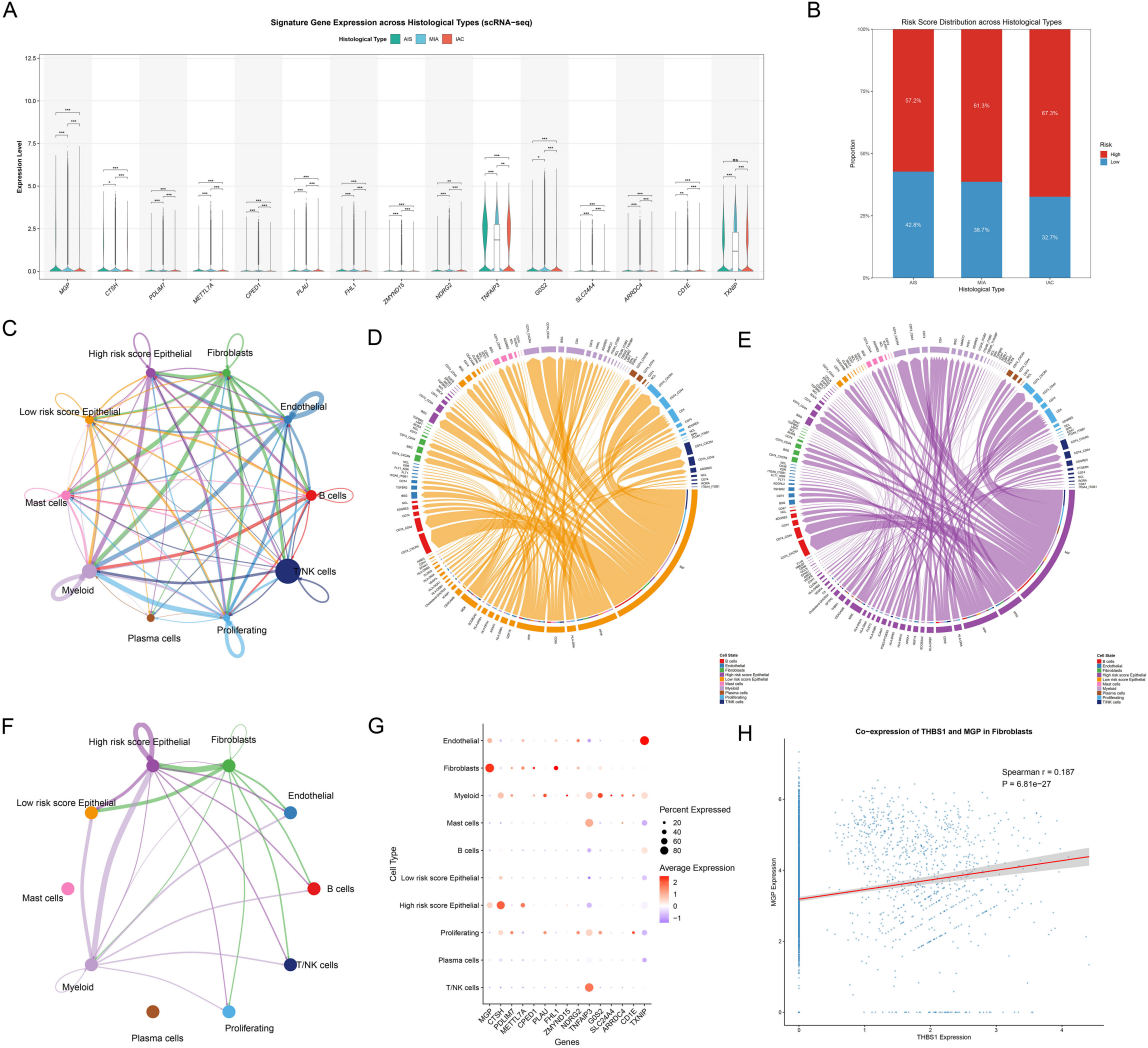
Single-cell validation of the CMA-related signature and THBS1-mediated communication during LUAD progression. **(A)** Violin plots showing the expression of the 15 signature genes across AIS, MIA, and IAC. **(B)** Stacked bar plot showing the proportions of high-risk and low-risk cells across histological stages. **(C)** CellChat analysis showing communication strengths among major cell populations after stratifying epithelial cells into high-risk and low-risk groups. **(D)** Representative communication pattern involving low-risk epithelial cells. **(E)** Representative communication pattern involving high-risk epithelial cells, highlighting the THBS1 pathway. **(F)** Directional signaling pattern of the THBS1 pathway across cell types. **(G)** Dot plot showing the expression distribution of the 15 signature genes across different cell types. **(H)** Scatter plot showing the correlation between THBS1 and MGP expression in Fibroblasts. ns, not significant; *P < 0.05, **P < 0.01, ***P < 0.001, ****P < 0.0001.

To further validate the reliability of this CMA-related signature at the single-cell level, we first compared the expression of the 15 signature genes across different histological stages. All 15 genes included in the model showed significant differences among AIS, MIA, and IAC, indicating that these key genes also displayed stable stage-dependent changes at the single-cell level, consistent with the bulk transcriptomic results (**Figure 9A**). This finding indicates that the signature is not only discriminative at the whole-sample level but also captures molecular heterogeneity during the AIS/MIA-to-IAC progression of LUAD at single-cell resolution.

We then compared the proportions of high-risk and low-risk cells across histological stages based on single-cell risk prediction results. As pathological stage progressed from AIS to MIA and IAC, the proportion of high-risk cells gradually increased, reaching 57.2%, 61.3%, and 67.3%, respectively, whereas the proportion of low-risk cells correspondingly declined from 42.8% to 38.7% and 32.7% (**Figure 9B**). These results indicate that the model also has good risk stratification ability at the single-cell level, and that the predicted high-risk state becomes progressively enriched with increasing pathological malignancy.

On this basis, we further focused on epithelial cells, stratified them into high-risk and low-risk groups, and constructed CellChat communication networks to assess differences in signaling intensities among cell types. Extensive communication links were observed among different cell populations, whereas high-risk and low-risk epithelial cells participated in the communication network in distinct ways. High-risk epithelial cells displayed more complex communication features with surrounding microenvironmental cells, suggesting that the risk state reflects not only intrinsic tumor cell transcriptional alterations but also reshaping of cross-cellular signaling exchange (**Figure 9C**).

Further comparison of the specific interaction pathways between high-risk or low-risk epithelial cells and other cell types showed that the THBS1 pathway was detected only in communication patterns associated with high-risk epithelial cells and was absent in the low-risk group. This finding suggests that THBS1 may represent a key pathway distinguishing microenvironmental interactions between high-risk and low-risk epithelial cells and provides an important clue for further focused analysis (**Figures 9D, E**).

Analysis of signal directionality across cell types further showed that fibroblasts served as the major signaling senders, acting on high-risk epithelial cells through the THBS1 pathway, indicating that the stromal microenvironment may play a more active role in driving the formation of malignant epithelial phenotypes (**Figure 9F**). Specifically, fibroblasts may enhance the responsiveness of high-risk epithelial cells to exogenous microenvironmental cues by secreting THBS1 and related ligands, thereby promoting the acquisition of a more invasive phenotype. At the same time, the higher expression of THBS1-related receptors or downstream genes in high-risk epithelial cells also suggests stronger responsiveness to fibroblast-derived signals, which may represent an important mechanism underlying their acquisition of a more aggressive phenotype.

We next examined the distribution of the 15 signature genes across different cell types. The dot plot showed that MGP, CTSH, PDLIM7, and PLAU were more prominently expressed in high-risk epithelial cells, suggesting that these genes may be more closely involved in the formation of the high-risk tumor epithelial state. In contrast, CD1E, TNFAIP3, and TXNIP were more predominantly distributed in Myeloid cells, indicating that part of the signature is closely associated with myeloid immune-related functions. In addition, several genes also showed detectable expression in fibroblasts, further suggesting that this signature is not driven solely by tumor cells but is jointly shaped by tumor cells and the microenvironment (**Figure 9G**).

Finally, we used MGP as an example to further assess its potential connection with THBS-related stromal signaling. Correlation analysis showed that THBS1 expression was significantly positively correlated with MGP expression in fibroblasts (Spearman r = 0.187, P < 0.001), suggesting that MGP may cooperate with THBS1-related stromal signals in tumor microenvironment remodeling (**Figure 9H**). Together with the communication analysis above, these findings suggest that THBS1-related fibroblast–tumor epithelial interactions may represent an important external driving force in the formation of the high-risk state.

Overall, single-cell analyses not only further validated the stability of this CMA-related signature during the AIS/MIA-to-IAC progression of LUAD but also revealed that the high-risk state is closely associated with more active microenvironmental interactions. In particular, computational inference by CellChat suggested that fibroblast-associated THBS1 signaling may represent a plausible stromal mechanism linked to the high-risk epithelial state.

### 3.10 External experiments validate the tumor-suppressive role of MGP in LUAD cells

**Figure 10:**
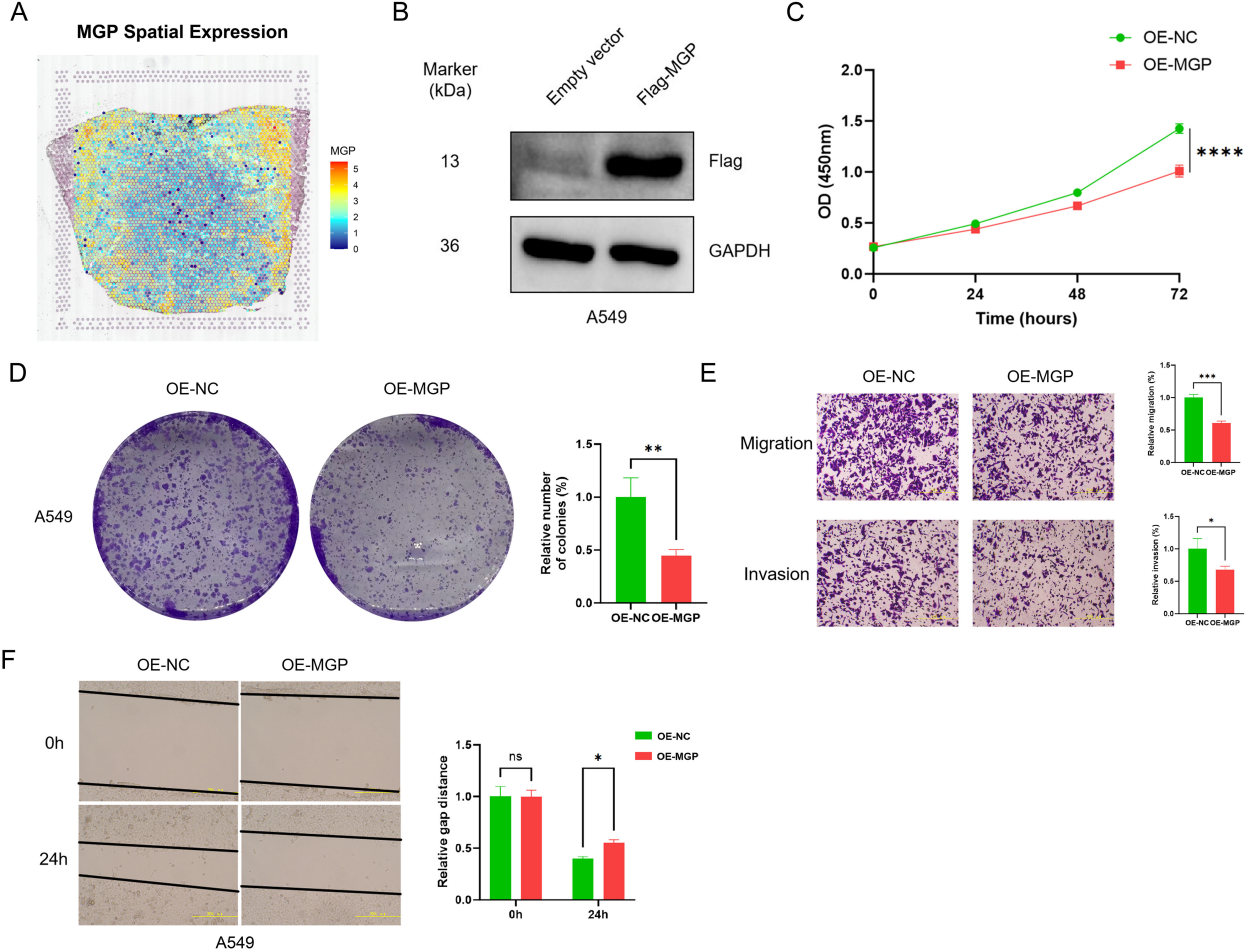
External experimental validation of MGP expression and function. **(A)** Spatial expression pattern of MGP in the tissue section. **(B)** Western blot analysis confirming successful overexpression of MGP. **(C)** CCK-8 assay showing that MGP overexpression inhibited cell proliferation. **(D)** Colony formation assay showing that MGP overexpression reduced clonogenic capacity. **(E)** Transwell assays showing that MGP overexpression suppressed cell migration and invasion. **(F)** Wound-healing assay showing that MGP overexpression inhibited migratory ability. ns, not significant; *P < 0.05, **P < 0.01, ***P < 0.001, ****P < 0.0001.

To further validate the biological significance of the core model gene MGP, we combined spatial expression analysis with *in vitro* functional experiments. MGP was prioritized for functional validation because it ranked highest in RSF variable importance and showed a relatively clear link to the stromal interaction pattern identified in the single-cell analysis. Spatial transcriptomic analysis showed that MGP was broadly distributed across the tissue section and exhibited relatively high expression signals in certain focal regions, indicating obvious spatial heterogeneity in tumor tissues (**Figure 10A**).

We then established an MGP overexpression model and confirmed it by Western blot. Compared with the empty vector control, transfection with Flag-MGP markedly increased the target band, whereas GAPDH remained stably expressed, indicating successful establishment of the MGP overexpression model and providing a reliable basis for subsequent functional assays (**Figure 10B**).

Regarding cell proliferation, CCK-8 assays showed that, compared with the OE-NC group, the proliferation curve of the OE-MGP group declined overall from 24 h onward, and the difference became most pronounced at 72 h, indicating that MGP overexpression significantly suppressed cell proliferation (P < 0.0001) (**Figure 10C**). Consistently, colony formation assays further demonstrated that clonogenic capacity was markedly reduced after MGP overexpression, with significantly fewer colonies than in the control group (P < 0.01), suggesting that MGP not only inhibits short-term cell viability but also impairs long-term clonogenic expansion (**Figure 10D**).

Regarding migration and invasion, Transwell assays showed that MGP overexpression simultaneously suppressed both migratory and invasive abilities. Compared with the OE-NC group, the OE-MGP group exhibited a marked reduction in the number of cells crossing the membrane, with a more pronounced decrease in migration (P < 0.001) and a significant suppression of invasion as well (P < 0.05) (**Figure 10E**). These findings indicate that MGP not only affects cell proliferation but also inhibits motility- and invasion-related phenotypes closely associated with tumor progression.

Wound-healing assays further verified the inhibitory effect of MGP on cell migration. No significant difference in wound width was observed between the two groups at 0 h, indicating comparable initial conditions. However, at 24 h, the OE-MGP group retained a wider wound gap and showed a higher relative gap distance than the control group, indicating impaired wound closure and reduced migratory ability (P < 0.05) (**Figure 10F**). This result is consistent with the Transwell assays and further supports the notion that MGP overexpression suppresses cell migration.

Taken together, the updated external experimental results further demonstrate that MGP overexpression significantly inhibits tumor cell proliferation, colony formation, migration, and invasion, supporting its tumor-suppressive role as a protective key gene in this study. These findings not only strengthen the biological credibility of the core model gene at the functional level but also further support its potential inhibitory role in LUAD progression.

### 3.11 Drug sensitivity analysis reveals distinct therapeutic vulnerabilities between risk groups

**Figure 11:**
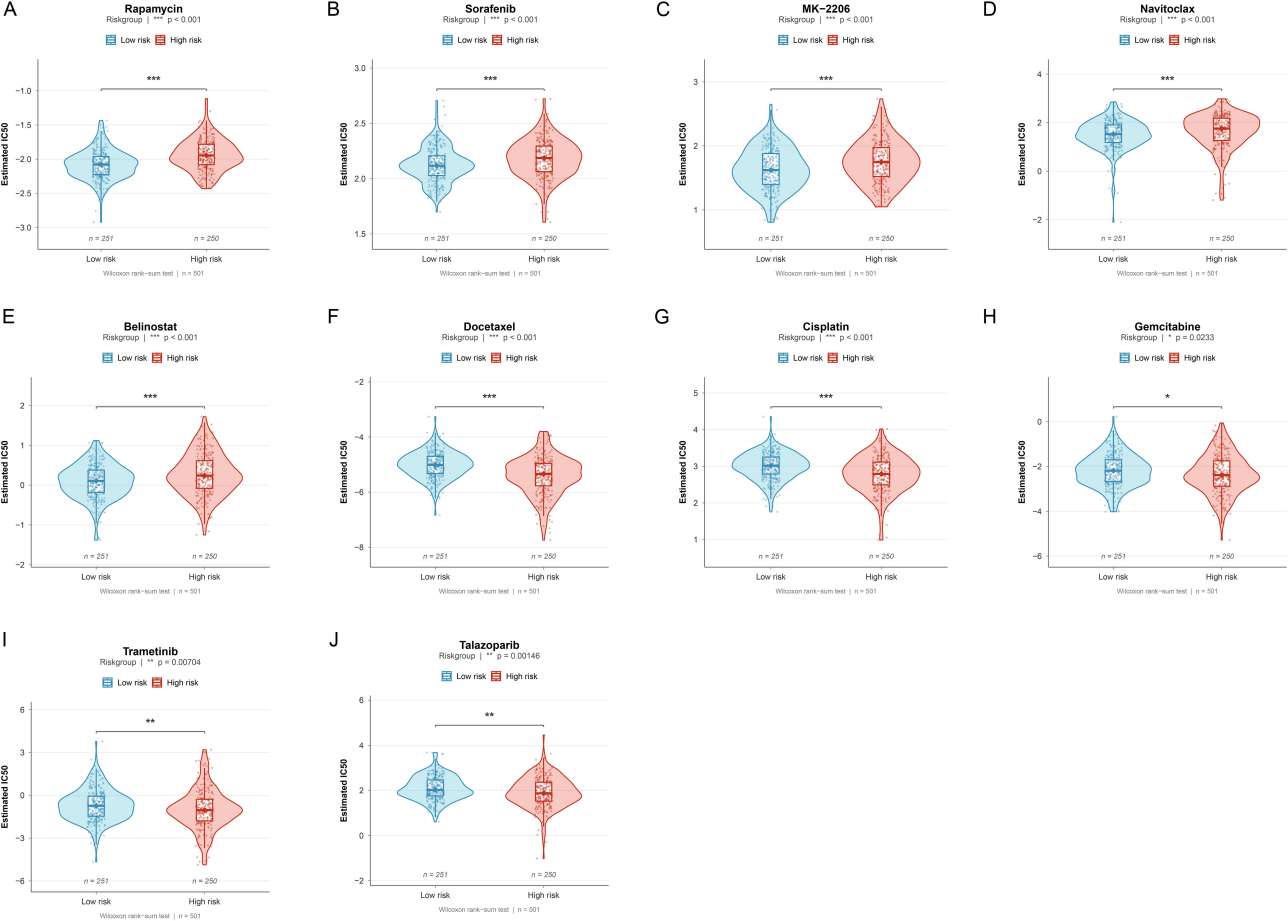
Drug sensitivity differences between the high-risk and low-risk groups. **(A)** Estimated IC50 of Rapamycin in the two risk groups. **(B)** Estimated IC50 of Sorafenib in the two risk groups. **(C)** Estimated IC50 of MK-2206 in the two risk groups. **(D)** Estimated IC50 of Navitoclax in the two risk groups. **(E)** Estimated IC50 of Belinostat in the two risk groups. **(F)** Estimated IC50 of Docetaxel in the two risk groups. **(G)** Estimated IC50 of Cisplatin in the two risk groups. **(H)** Estimated IC50 of Gemcitabine in the two risk groups. **(I)** Estimated IC50 of Trametinib in the two risk groups. **(J)** Estimated IC50 of Talazoparib in the two risk groups. ns, not significant; *P < 0.05, **P < 0.01, ***P < 0.001.

To further explore the potential utility of this CMA-related risk signature in treatment stratification, we compared the predicted sensitivity of the high-risk and low-risk groups to multiple representative anticancer drugs. The results showed that different risk states corresponded to clearly distinct drug response profiles, suggesting that this signature may not only stratify prognosis but also provide clues for individualized therapeutic strategies (**Figure 11**).

First, for Rapamycin, Sorafenib, MK-2206, Navitoclax, and Belinostat, the estimated IC50 values were all significantly higher in the high-risk group than in the low-risk group, indicating lower sensitivity in high-risk patients and suggesting that low-risk patients may derive greater benefit from these treatments. Differences for Rapamycin, Sorafenib, MK-2206, Navitoclax, and Belinostat all reached statistical significance (all P < 0.001), suggesting that the high-risk state may be associated with relative resistance to therapies related to mTOR inhibition, multi-kinase inhibition, AKT inhibition, apoptosis regulation, and epigenetic modulation (**Figures 11A–E**). Mechanistically, these drugs cover mTOR pathway inhibition, RAF/VEGFR/PDGFR-related multi-target signaling, PI3K/AKT signaling, BCL2/BCL-XL-dependent apoptosis regulation, and HDAC-mediated epigenetic regulation, implying that low-risk patients may retain higher therapeutic sensitivity along these targeted and regulatory axes.

By contrast, for Docetaxel, Cisplatin, Gemcitabine, Trametinib, and Talazoparib, the estimated IC50 values were higher in the low-risk group than in the high-risk group, indicating that high-risk patients may be more sensitive to these agents. Among them, Docetaxel and Cisplatin showed the most significant differences (both P < 0.001), Gemcitabine also reached statistical significance (P = 0.0233), and Trametinib and Talazoparib likewise showed significant differences (P = 0.00704 and P = 0.00146, respectively) (**Figures 11F–J**). These drugs represent microtubule stabilization, DNA crosslinking damage, antimetabolite therapy, MAPK/ERK pathway inhibition, and PARP-mediated DNA repair targeting, respectively, suggesting that although the high-risk group had poorer overall prognosis, it might exhibit greater vulnerability to certain classical cytotoxic agents, MAPK pathway inhibitors, and DNA repair-related therapies.

Overall, the high-risk and low-risk groups exhibited two distinct treatment response patterns. The low-risk group appeared more likely to benefit from Rapamycin, Sorafenib, MK-2206, Navitoclax, and Belinostat, representing targeted, apoptosis-regulating, and epigenetic therapies, whereas the high-risk group appeared more likely to benefit from Docetaxel, Cisplatin, Gemcitabine, Trametinib, and Talazoparib, representing microtubule inhibition, DNA damage, antimetabolite therapy, MAPK pathway inhibition, and DNA repair deficiency-related targeting. These results indicate that the CMA-related risk signature not only reflects survival heterogeneity in LUAD, but also reveals potential therapeutic vulnerabilities associated with different risk states, thereby providing a basis for risk-adapted precision treatment.

## 4 Discussion

By integrating single-cell transcriptomics, spatial transcriptomics, bulk transcriptomics, machine learning modeling, and *in vitro* functional experiments, this study systematically characterized the dynamic changes of CMA-related features during the continuous progression of LUAD from AIS and MIA to IAC and established a CMA-related signature with stable prognostic stratification ability. Previous single-cell and spatial studies of the AIS/MIA-to-IAC progression of LUAD have already shown that the transition from AIS to IAC is not simply a process of tumor cell expansion, but rather one accompanied by continuous remodeling of tumor cell states, immune ecology, and spatial neighborhoods (32, 33). Zhu et al. also demonstrated clear multicellular compositional changes and spatial reprogramming across AIS, MIA, and IAC in the early LUAD system corresponding to GSE189357/GSE189487 (4). Our findings are consistent with this overall framework but extend it one step further by specifically anchoring such early progression-related heterogeneity to CMA-related programs and showing that the most prominent changes do not occur at the level of average tumor expression, but are concentrated in specific cellular compartments, particularly the myeloid compartment.

The role of CMA in cancer has long been recognized as highly context-dependent (34). Previous studies have shown that CMA can exert either tumor-promoting or tumor-suppressive effects depending on tumor type and biological context, while aberrant upregulation of LAMP2A is often associated with tumor survival, therapeutic resistance, and poor prognosis (35, 36). In NSCLC, Ichikawa et al. reported that elevated LAMP2A expression was associated with worse survival and platinum resistance, suggesting that CMA activation may contribute to lung cancer progression and therapeutic resistance (12). Compared with these studies, the major advance of the present work does not lie in merely confirming that CMA is “related to tumors,” but rather in demonstrating that CMA during the AIS/MIA-to-IAC progression of LUAD is not a globally synchronized event. Instead, it appears to be a localized process driven by remodeling of specific cellular states. In particular, we found that myeloid cells displayed overall high CMA activity and underwent clear subtype replacement during disease progression, suggesting that CMA-related transcriptional programs are likely embedded in tumor-associated myeloid remodeling rather than representing only an intrinsic metabolic adaptation of tumor epithelial cells.

This also helps explain why the candidate genes ultimately identified in this study did not appear randomly scattered, but were instead enriched in processes such as phagocytosis, myeloid immune activation, lipid transport, cell-surface immune interaction, and pattern recognition (37, 38). Recent single-cell studies have suggested that the formation of a tumor-promoting microenvironment is often not determined by changes in the abundance of a single immune cell type, but rather jointly shaped by specific myeloid and fibroblast states (39, 40). In early LUAD, we observed enrichment of cDC2 and APOE+ lipid-associated TAMs in IAC, whereas FABP4+ metabolic TAMs and immature neutrophils were relatively decreased (41–43). These findings suggest that the myeloid compartment does not simply expand but instead shifts toward a more organized state favorable for sustained tumor growth and invasion. Compared with many studies that directly build models based only on bulk differential expression analysis, our study combined tumor vs normal differential expression analysis with WGCNA at the bulk level to identify candidate genes before entering machine learning modeling. This strategy constrains the upstream source of the signature by both disease-related transcriptional abnormalities and CMA-related co-expression patterns, thereby enhancing its biological coherence with subsequent immune microenvironment and cell-cell communication findings.

At the level of microenvironmental interactions, one of the most novel findings of this study is that the THBS1 pathway was detected only in communication patterns associated with high-risk epithelial cells, and that the direction of signaling was mainly from fibroblasts to high-risk epithelial cells. Previous studies have suggested that THBS1 is not merely a stromal marker, but is closely associated with stromal activation, immune suppression, and poor prognosis (44, 45). In colorectal cancer, high THBS1 expression has been linked to stromalization, immune suppression, and unfavorable outcome, and more recent spatial multi-omics studies have shown that specific CAF subsets can interact with cancer cells through axes such as THBS1-CD47, contributing to immune evasion and tumor progression (46). Our findings are consistent with these studies in direction, but place this phenomenon earlier, at the stage of LUAD progression, and suggest that fibroblasts may be involved in shaping the high-risk epithelial state rather than merely accompanying the emergence of advanced tumors. This transforms the “high-risk epithelial state” from an abstract transcriptional label into a biologically interpretable process with a definable microenvironmental source.

Functional validation of MGP provides key support for the above interpretations. Although previous studies have mentioned MGP in the context of CAF-related gene expression changes in NSCLC, its functional role in lung cancer has remained unclear (47, 48). Our results showed that MGP not only ranked highest in RSF variable importance, but that its overexpression significantly suppressed lung cancer cell proliferation, colony formation, migration, and invasion, suggesting that it is more likely to be a protective functional gene rather than merely an associated marker. Importantly, this is not contradictory to the prognostic model, because the RSF-based signature reflects the combined contribution of multiple genes rather than requiring all components to act in the same biological direction. At the same time, the remaining signature genes also suggest that this model captures multiple biological processes rather than a single molecular axis. For example, several genes in the signature, including PLAU, CTSH, and PDLIM7, are associated with invasion-related or cytoskeleton-associated programs, whereas others, such as TXNIP, G0S2, ARRDC4, and NDRG2, are more closely linked to cellular stress responses, metabolic adaptation, or growth restraint (49–51). In addition, CD1E and TNFAIP3 suggest that the signature also contains an immune- and myeloid-related component. This is consistent with the overall framework of our study, namely that formation of the high-risk state does not simply reflect enhancement of tumor-promoting programs but may also involve weakening of molecular constraints that normally maintain homeostasis or suppress malignant phenotypes (52). In addition, drug sensitivity analysis further suggested that different risk states correspond to distinct therapeutic vulnerabilities. The low-risk group appeared more sensitive to therapies related to mTOR, AKT, multi-target kinase inhibition, apoptosis regulation, and HDAC modulation, whereas the high-risk group appeared more sensitive to classical cytotoxic agents, MEK inhibition, and PARP-related strategies (53–55). These drug sensitivity results were derived from the risk model and should therefore be interpreted as signature-level predictive findings. These findings indicate that high risk does not imply universal treatment resistance but may instead reflect a tumor state more dependent on high proliferation, replication stress, and DNA damage response.

This study still has several limitations. First, although the model was validated in two external cohorts, further confirmation in prospective clinical cohorts is still needed. Second, aside from MGP, the specific functions of the remaining key genes in different cellular compartments remain to be experimentally clarified. Third, the functional validation of MGP was performed in only one LUAD cell line, and no *in vivo* experiments were included. Therefore, the current experimental findings should be interpreted as preliminary functional support, and further validation in additional LUAD cell lines and animal models will be needed in future studies. Fourth, although THBS1-related communication suggests that fibroblasts may actively drive the high-risk epithelial state, this conclusion is currently based mainly on computational inference and will require further confirmation through co-culture, blocking experiments, or spatial *in situ* validation. Finally, the drug sensitivity analysis was based on estimated IC50 values and is therefore more suitable for generating therapeutic hypotheses than for directly guiding clinical drug selection. Nevertheless, the strength of this study lies in the high degree of directional consistency across different levels of data, indicating that this CMA-related signature can not only robustly predict prognosis in LUAD, but also reflect key biological events during early tumor evolution, including myeloid remodeling, metabolic reprogramming, immune imbalance, and enhanced microenvironmental communication.

## 5 Conclusion

This study systematically characterized the heterogeneity of CMA-related features during the progression of LUAD from AIS to IAC and established a robust 15-gene prognostic signature. This signature showed favorable prognostic stratification ability in both the training and external validation cohorts and was closely associated with metabolic reprogramming, immune microenvironment remodeling, and altered cell-cell communication. Further analyses suggested that THBS1-related fibroblast–epithelial communication may be associated with the formation of the malignant phenotype in high-risk epithelial cells, whereas the core gene MGP exhibited a clear tumor-suppressive role *in vitro*. Overall, this study provides new evidence for understanding the molecular evolution and risk stratification of early LUAD.

## Data Availability

The original contributions presented in the study are included in the article/**Supplementary Material**. Further inquiries can be directed to the corresponding authors.
