# Supplementary material for: Decoding early lung adenocarcinoma progression by single-cell and spatial transcriptomics reveals a CMA-related prognostic signature
Source: Front Immunol. 2026 Jul 9;17:1875096. doi: 10.3389/fimmu.2026.1875096 (PMC13391922; doi:10.3389/fimmu.2026.1875096)
Supplement: Supplementary file 1 [file Table1.docx]

Supplementary Figure S1. Determination of the soft-thresholding power for WGCNA.


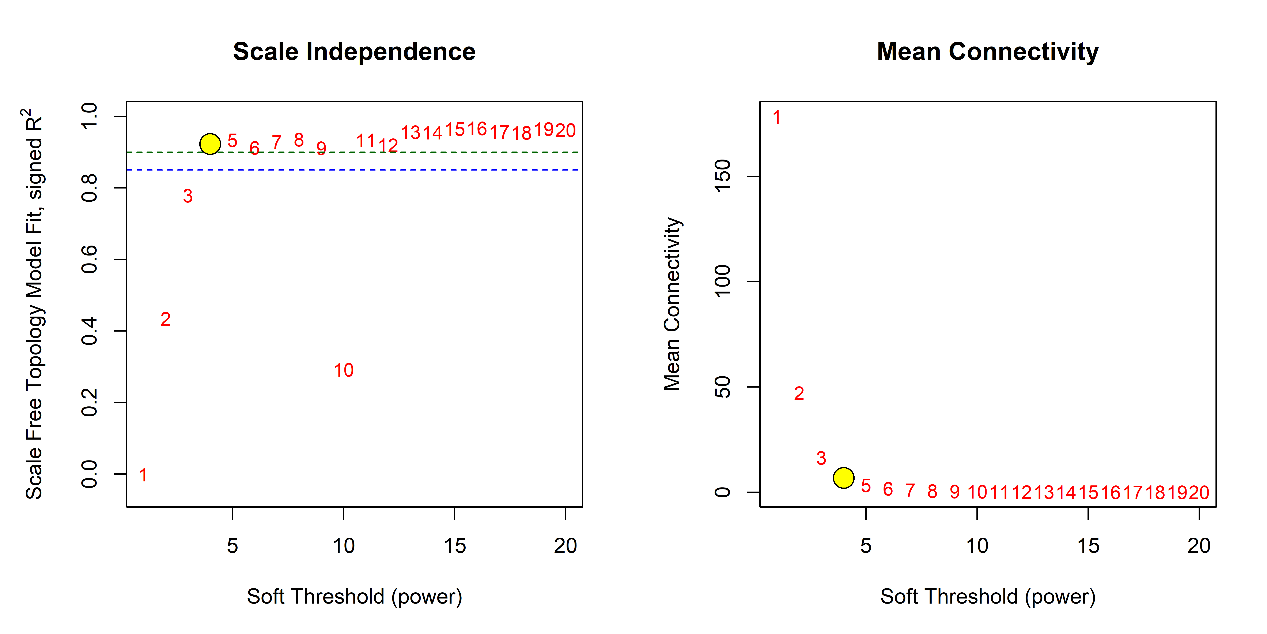


(Left) Scale-free topology model fit index (signed R²) plotted against candidate soft-thresholding powers. The blue and green dashed lines indicate R² = 0.85 and 0.90, respectively. (Right) Mean network connectivity across candidate powers. The selected power (β = 4) is highlighted with a yellow circle; at this value, signed R² = 0.923 and mean connectivity = 6.83.
