# Supplementary material for: Decoding early lung adenocarcinoma progression by single-cell and spatial transcriptomics reveals a CMA-related prognostic signature
Source: Front Immunol. 2026 Jul 9;17:1875096. doi: 10.3389/fimmu.2026.1875096 (PMC13391922; doi:10.3389/fimmu.2026.1875096)
Supplement: Supplementary file 2 [file Table2.docx]

**Supplementary Table S1. CMA-related gene set used for CMA score calculation**

| **Category** | **Gene symbols** | **Role in CMA score** |
| --- | --- | --- |
| Effector genes (n = 26) | AMBRA1, ATG3, ATG4A, ATG4B, ATG4C, ATG4D, ATG5, ATG7, ATG9A, ATG9B, ATG10, ATG12, ATG16L1, ATG16L2, MAP1LC3A, MAP1LC3B2, GABARAP, GABARAPL1, GABARAPL2, NBR1, OPTN, RAB24, BNIP3, SQSTM1, BNIP3L, CALCOCO2 | Core execution machinery |
| Positive regulators (n = 43) | ULK1, BECN1, ULK2, BAD, BAK1, BAX, BID, CDKN1B, CDKN2A, CXCR4, DAPK1, DRAM1, EIF2AK3, FAS, HIF1A, HTT, HGS, NRBF2, NFKB1, PIK3CG, PIK3C3, PIK3R4, PRKAA1, PRKAA2, PRKAG2, PTEN, RAB7A, RB1, STK40, TGFB1, TGM2, TMEM74, TP53, TP73, UVRAG, VPS8, VPS11, VPS18, VPS26A, VPS33A, VPS35, WDR45B, RGS10 | Positive regulatory component |
| Negative regulators (n = 15) | AKT1, BCL2, BCL2L1, CASP3, CASP8, CLN3, EIF4G1, FADD, HDAC1, IGF1, MAPK8, MAPK14, RRAGC, RPS6KB1, SNCA | Negative regulatory component |

Note:
CMA score = (2 × sum of effector gene expression + sum of positive regulator gene expression − sum of negative regulator gene expression) / 84.
