# Supplementary material for: Decoding early lung adenocarcinoma progression by single-cell and spatial transcriptomics reveals a CMA-related prognostic signature
Source: Front Immunol. 2026 Jul 9;17:1875096. doi: 10.3389/fimmu.2026.1875096 (PMC13391922; doi:10.3389/fimmu.2026.1875096)
Supplement: Supplementary file 3 [file Table3.docx]

**Supplementary Table S2. Baseline demographic and clinicopathological characteristics of the TCGA-LUAD, GSE31210, and GSE50081 cohorts**

| **Characteristic** | **TCGA-LUAD** | **GSE31210** | **GSE50081** |
| --- | --- | --- | --- |
| **Sample size, n** | 501 | 226 | 127 |
| **Age, years, median (range)** | 66 (33–88) | 61 (30–76) | 69.92 (40–85) |
| **Sex, n (%)** |  |  |  |
| Female | 271 (54.1%) | 121 (53.5%) | 62 (48.8%) |
| Male | 230 (45.9%) | 105 (46.5%) | 65 (51.2%) |
| **Smoking status, n (%)** |  |  |  |
| Never-smoker / Never | N/A | 115 (50.9%) | 23 (18.1%) |
| Ever-smoker / Current / Ex-smoker | N/A | 111 (49.1%) | 92 (72.4%) |
| Unable to determine | N/A | N/A | 12 (9.4%) |
| **Stage, n (%)** |  |  |  |
| I / IA / IB | 270 (53.9%) | 168 (74.3%) | 92 (72.4%) |
| II / IIA / IIB | 119 (23.8%) | 58 (25.7%) | 35 (27.6%) |
| III | 79 (15.8%) | 0 | 0 |
| IV | 25 (5.0%) | 0 | 0 |
| Missing / unreported | 8 (1.6%) | 0 | 0 |
| **OS events, n (%)** | 180 (35.9%) | 35 (15.5%) | 51 (40.2%) |
| Age is presented as median (range). Smoking information was available for GSE31210 and GSE50081 but was not consistently available in the TCGA-LUAD cohort used in this study. Stage categories were harmonized across datasets for summary display, with GSE50081 stage IA/IB grouped as stage I and stage IIA/IIB grouped as stage II. | | | |
